# Parkinson disease-associated Leucine-rich repeat kinase regulates UNC-104-dependent axonal transport of Arl8-positive vesicles in *Drosophila*

**DOI:** 10.1016/j.isci.2022.105476

**Published:** 2022-11-02

**Authors:** Tsuyoshi Inoshita, Jun-Yi Liu, Daisuke Taniguchi, Ryota Ishii, Kahori Shiba-Fukushima, Nobutaka Hattori, Yuzuru Imai

**Affiliations:** 1 Department of Neurodegenerative and Demented Disorders, Juntendo University Graduate School of Medicine, 2-1-1 Hongo, Bunkyo-ku, Tokyo 113-8421, Japan; 2 Department of Neurology, Juntendo University Graduate School of Medicine, 2-1-1 Hongo, Bunkyo-ku, Tokyo 113-8421, Japan; 3 Department of Research for Parkinson’s Disease, Juntendo University Graduate School of Medicine, 2-1-1 Hongo, Bunkyo-ku, Tokyo 113-8421, Japan; 4 Department of Drug Development for Parkinson’s Disease, Juntendo University Graduate School of Medicine, 2-1-1 Hongo, Bunkyo-ku, Tokyo 113-8421, Japan; 5 Neurodegenerative Disorders Collaborative Laboratory, RIKEN Center for Brain Science, 2-1-Hirosawa, Wako-shi, Saitama 351-0198, Japan

**Keywords:** Biological sciences, Genetics, Neuroscience, Cell biology

## Abstract

Some Parkinson’s disease (PD)-causative/risk genes, including the PD-associated kinase leucine-rich repeat kinase 2 (LRRK2), are involved in membrane dynamics. Although LRRK2 and other PD-associated genes are believed to regulate synaptic functions, axonal transport, and endolysosomal activity, it remains unclear whether a common pathological pathway exists. Here, we report that the loss of Lrrk, an ortholog of human LRRK2, leads to the accumulation of the lysosome-related organelle regulator, Arl8 along with dense core vesicles at the most distal boutons of the neuron terminals in *Drosophila*. Moreover, the inactivation of a small GTPase Rab3 and altered Auxilin activity phenocopied Arl8 accumulation. The accumulation of Arl8-positive vesicles is UNC-104-dependent and modulated by PD-associated genes, Auxilin, VPS35, RME-8, and INPP5F, indicating that VPS35, RME-8, and INPP5F are upstream regulators of Lrrk. These results indicate that certain PD-related genes, along with LRRK2, drive precise neuroaxonal transport of dense core vesicles.

## Highlights

• LRRK mutations lead to Arf-like GTPase Arl8 accumulation at presynapses in *Drosophila*
• Arl8 accumulation is associated with dense core vesicles and UNC-104 dependent
• PD-related genes VPS35, RME-8, Auxilin, and INPP5F are involved in Arl8 dynamics

## Graphical abstract

**Figure:**
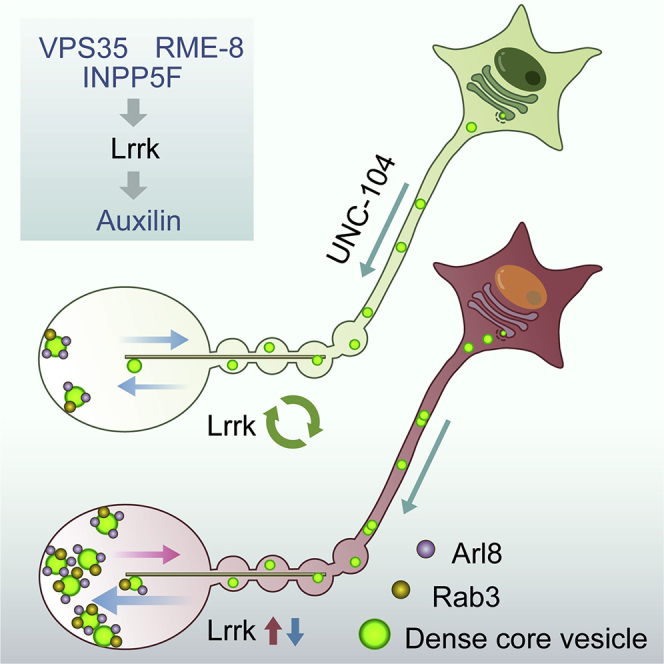

## Introduction

Parkinson disease (PD) is a neurodegenerative disorder characterized primarily by movement disturbance. Although the etiology of PD is still unknown, 5-10% of PD is a monogenic form with Mendelian inheritance (Deng 2018; Inoshita 2018). To date, more than 20 genes have been identified, some of which are involved in membrane trafficking (Inoshita 2018). For example, one of the retromer components VPS35 [also known as *PARK17* in online mendelian inheritance in man (OMIM) (https://www.omim.org)] and a J-domain containing the protein RME-8 (*PARK21*) work with the WASH complex to regulate vesicle budding^1^^,^^2^^,^^3^; Auxilin (Aux, *PARK19*) regulates clathrin-mediated endocytosis from the plasma membrane^4^ and budding from *trans*-Golgi^5^^,^^6^^,^^7^; Synaptojanin1 (*PARK20*), which is a phosphoinositide 4,5-phosphatase, is involved in synaptic vesicle (SV) endocytosis.^8^ A phosphoinositide 4-phosphatase INPP5F, which has been identified as a PD risk gene in a genome-wide association study,^9^ functions as an effector of Rab5 to control endocytosis^10^^,^^11^ and phagocytosis,^12^ and regulates docking and release of insulin granules together with Rab3.^13^ The *Rab29/Rab7L1* gene is located at the *PARK16* locus^14^ and is a possible PD causative gene.^15^ A study showed that Rab29-deficient mice developed defects in kidney proximal tubule cells, similar to LRRK2 (*PARK8*)-deficient mice.^16^ Moreover, overexpression of Rab29 results in the activation of LRRK2 kinase.^17^^,^^18^ However, another study reported no alteration in LRRK2 activity in Rab29-deficient mice.^19^ Thus, it is controversial whether Rab29 is a regulatory molecule of LRRK2.

Missense mutations of *LRRK2* have been identified in late-onset hereditary PD^20^^,^^21^ and sporadic PD worldwide.^22^ Thus, *LRRK2* is considered one of the most important genes in PD research. Increased LRRK2 kinase activity is observed in many pathogenic mutations.^23^^,^^24^^,^^25^ LRRK2 phosphorylates several Rab GTPases, including Rab3, Rab8, Rab10, and Rab35.^26^ Rab GTPases phosphorylated by LRRK2 are activated by the dissociation of Rab GDP dissociation inhibitors.^23^

Studies in neurons and glial cells have shown various functions of LRRK2, including microtubule modification,^27^^,^^28^^,^^29^^,^^30^ primary cilia formation,^26^^,^^31^^,^^32^ membrane elongation,^33^ and autophagosome transport^34^ via the microtubule-dependent motor adapter JIP4. On the other hand, among the PD causative/risk genes involved in membrane transport, VPS35,^35^^,^^36^ Auxilin (Aux),^37^ Synaptojanin1,^38^^,^^39^ and Rab29^17^^,^^18^ have been reported as molecules involved in LRRK2 signaling. However, these studies have only analyzed the molecular relationship between the two and do not provide a panoramic view of LRRK2 signaling.

Previous *C*. *elegans* studies have reported that the loss of *lrk-1*, an *LRRK2* ortholog, leads to missorting of neuronal axonal trafficking,^40^ and the sorting of synaptic molecules from *trans*-Golgi by LRK-1 is mediated by clathrin, AP3, and Unc-16/SYD (ortholog of JIP 3 and JIP4).^41^ Another genetic study using *C*. *elegans* observed that LRK-1 regulates proper extension of the axonal terminals and that *GLO-1*, an ortholog of *Rab29* in *C*. *elegans*, and *AP3* are located upstream and downstream of *lrk-1*, respectively.^16^

*Drosophila* has a single LRRK2 ortholog, Lrrk, and age-dependent neurodegeneration is observed upon the introduction of pathogenic mutations.^42^ On the other hand, loss of Lrrk is associated with abnormal presynaptic activity.^35^^,^^43^^,^^44^ The above-mentioned studies in *C*. *elegans* and *Drosophila* suggest that LRRK2 is involved in the transport of molecules required for presynaptic activity.

A current study reports that Lrrk mutations in *Drosophila* result in the accumulation of Arf-like GTPase Arl8, which is involved in the transport of lysosome-related organelles,^45^ and dense core vesicles (DCVs) in distal synaptic boutons. Based on this phenotype, we conducted a genetic interaction analysis between Lrrk and known PD causative/risk genes that are involved in membrane dynamics, providing a landscape of LRRK2 signaling. Our study suggests that Aux, RME-8, VPS35, and INPP5F are involved in LRRK2 signaling and contribute to the precise transport of proteins associated with DCVs to the presynapses.

## Results

### Mutations in leucine-rich repeat kinase lead to Arl8 accumulation at synaptic terminals

**Figure 1:**
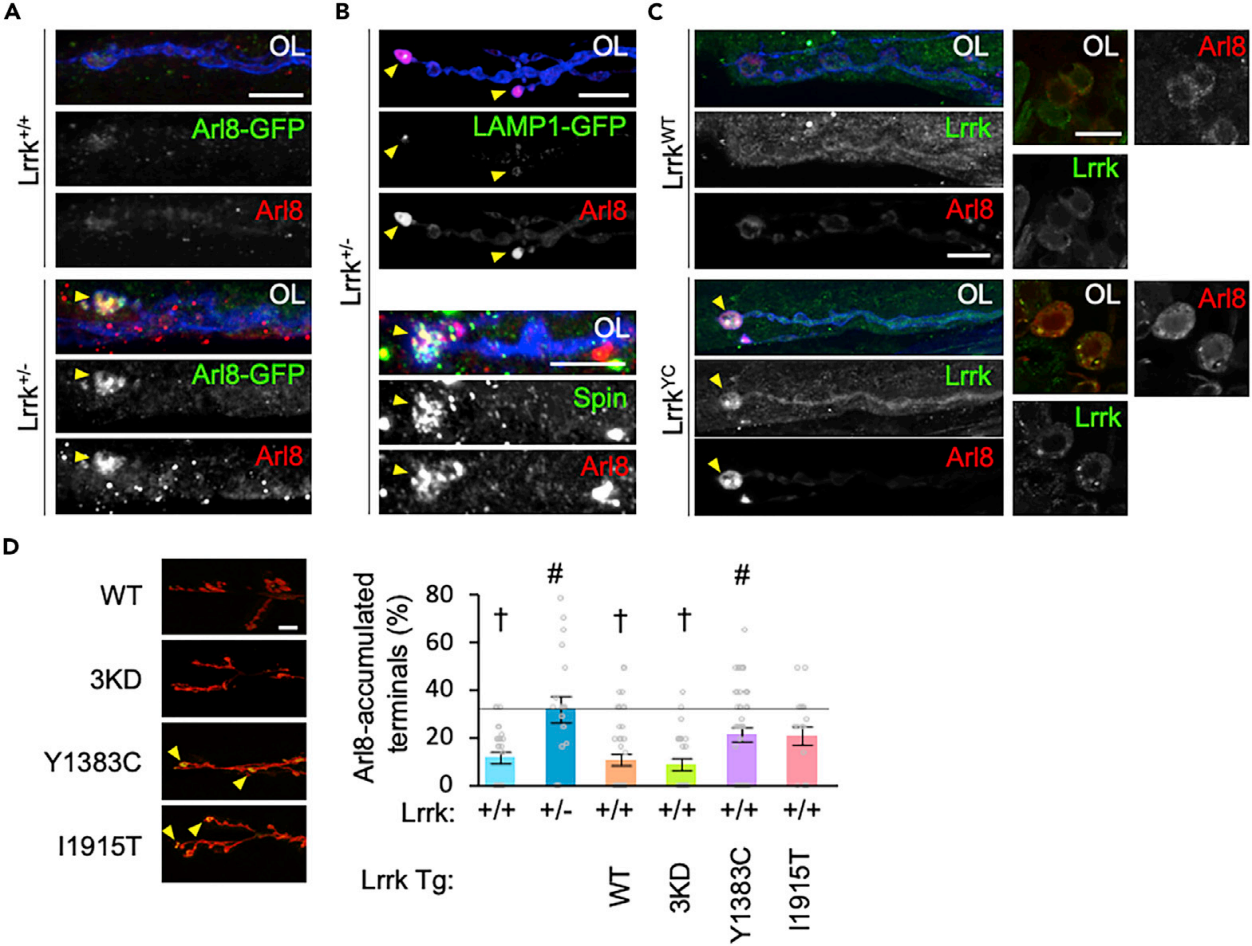
Mutations in Lrrk causes the accumulation of Arl8 in the synaptic boutons of the neuromuscular junction (NMJ) (A) Distribution of Arl8 in the NMJ synaptic boutons of the indicated genotypes. Arl8 and synaptic boutons in the NMJ were visualized using Arl8-GFP knock-in (green) along with anti-Arl8 (red) and DyLight649-conjugated anti-horseradish peroxidase (HRP, blue). Arrowheads indicate Arl8 accumulations in the most distal synaptic boutons. Each top image shows overlay (OL), while the middle and bouton images show single-channel images in grayscale. Scale bar, 10 μm. (B) Localization of Arl8 along with LAMP1 or Spinster in *Lrrk*^*+/−*^ boutons. (C) Distribution of Arl8 and Lrrk in the synaptic boutons at the NMJs (left) and cell bodies in the ventral ganglia (right) overexpressing Lrrk WT or Y1383C (YC). Scale bars, 10 μm. (D) Distribution of Arl8 in the synaptic boutons at the NMJs overexpressing Lrrk WT, 3KD, Y1383C, or I1915T. Arrowheads indicate Arl8 accumulations in the most distal synaptic boutons. Scale bar, 20 μm. Graph represents mean ± SEM (n = 19–46 NMJs in 6–12 flies). #p < 0.05 vs. Lrrk^+/+^; †p < 0.05 vs. Lrrk^+/−^ by Dunnett’s test. See also Figure S1.

We have previously reported that Lrrk and VPS35 cooperate in the endocytosis of presynaptic SVs.^35^ Both Lrrk and VPS35 mutants exhibit cisternal structures at presynapses, suggesting abnormal membrane dynamics.^35^^,^^44^ To further characterize these presynaptic phenotypes, we systematically stained the presynapses of *Lrrk* loss-of-function (LOF) flies with marker antibodies of each organelle (Figure S1). We found that Arl8 was accumulated at the presynaptic terminal boutons of motor neurons in *Lrrk*^*+/−*^ and *Lrrk*^*−/−*^ (Figure 1A). Arl8 is involved in the co-transport of SV and active zone proteins in presynaptic lysosome-related vesicles.^45^^,^^46^ Arl8-positive presynaptic structures were partially positive for the lysosome-related proteins LAMP1 and Spinster (Figure 1B). Overexpression of Lrrk Y1383C, which corresponds to the human LRRK2 pathogenic mutant Y1699C, also produced Arl8 aggregates at the terminal boutons, while wild-type (WT) Lrrk overexpression did not (left in Figure 1C). Lrrk Y1383C, but not WT, also formed puncta with Arl8 both in the terminal boutons and the cell bodies of motor neurons (Figure 1C). These puncta contained Rab7 and partly lysosomes (Figures S1A and S1B). On the other hand, overexpression of kinase-dead Lrrk (3KD), like WT, showed no Arl8 accumulation, while I1915T (corresponding to the human LRRK2 I2020T) tended to accumulate it (Figure 1D). There was no presynaptic accumulation of other organelle makers, which include Rabenosyn-5 (early endosomes), Calnexin 99A (ER), Hrs (multivesicular body), Galactosyltransferase (*trans*-Golgi), and an autophagy marker, Ref(2)P (Figure S1C).

### Rab3 inactivation promotes Arl8 accumulation

**Figure 2:**
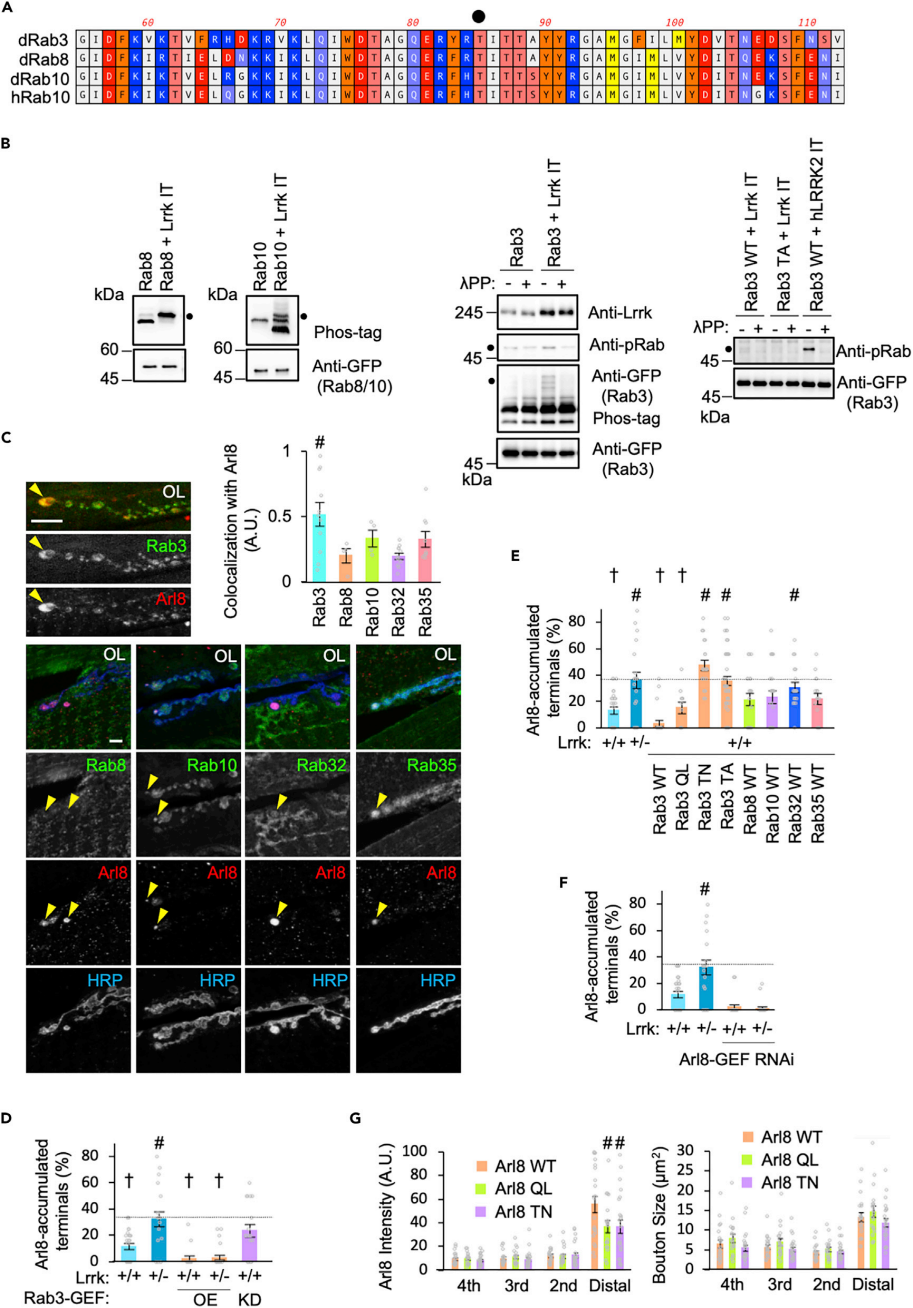
Altered Rab3 activity causes Arl8 accumulation (A) Sequence alignment of human Rab10 (hRab10) and *Drosophila* Rab (dRab) proteins. Dot indicates hRab10 T72 phosphorylated by human LRRK2 and the corresponding residues in dRab proteins. (B) Phosphorylation of Rab3, Rab8, and Rab10 by Lrrk I1915T (Lrrk IT) or human LRRK2 I2020T (hLRRK2 IT). Phosphorylation of Rab proteins was analyzed with (+) or without (−) Lambda protein phosphatase (λPP) treatment by phos-tag or anti-phospho-Rab. Dots indicate *Lrrk*-dependent phosphorylation. (C) Rab3 is condensed in Arl8 aggregates caused by *Lrrk* loss. Distribution of Rab proteins in Arl8 aggregate-positive synaptic boutons was analyzed. Arrowheads indicate Arl8 accumulations. Graph represents the ratio of the intensity of Rab signals in the Arl8 aggregate-positive boutons to their averaged intensity in three neighboring boutons in *Lrrk*^*+/−*^. Scale bar, 10 μm #p < 0.05 vs. Rab8 by Dunnett’s test. (D) Reduced Rab3-GEF activity causes Arl8 accumulation, while Rab3-GFP overexpression suppresses it. Graph represents mean ± SEM (n = 16–33 NMJs in 4–9 flies). #p < 0.05 vs. Lrrk^+/+^; †p < 0.05 vs. Lrrk^+/−^ by Dunnett’s test. (E) Reduced Rab3 GTPase activity causes Arl8 accumulation. Graph represents mean ± SEM (n = 18–45 NMJs in 5–12 flies). #p < 0.05 vs. Lrrk^+/+^; †p < 0.05 vs. Lrrk^+/−^ by Dunnett’s test. (F) Reduced Arl8 GTPase activity due to Ar8-GEF knockdown suppresses presynaptic Arl8 accumulation. Graph represents mean ± SEM (n = 19–26 NMJs in 6–7 flies on an *elav-GAL4* genetic background). #p < 0.05 vs. Lrrk^+/+^ by Dunnett’s test. (G) Ectopic expression of Arl8 WT, but not Arl8 Q75L or T34N, promotes presynaptic Arl8 accumulation. Left and right graphs indicate Arl8 intensity in each bouton and each bouton size, respectively. The most distal, second, third, and fourth boutons of the terminal are labeled in Figure S2D. Graph represents mean ± SEM (n = 21–26 NMJs in 6–7 flies on an *elav-GAL4* genetic background). #p < 0.05 vs. Arl8 WT by Dunnett’s test. See also Figure S2.

Rab3, Rab8, Rab10, and Rab35 are known to be substrates of mammalian LRRK2.^26^^,^^47^ The amino acid sequences around the phosphorylation residue (T73) of human Rab10 by mammalian LRRK2 are highly conserved among *Drosophila* Rab3, Rab8, and Rab10 (Figure 2A). Lrrk overexpression also phosphorylated *Drosophila* Rab3, Rab8, and Rab10, although the efficiency of Rab3 phosphorylation was weaker than the other two (Figure 2B). Arl8 positive structures are well co-localized with Rab3 and weakly co-localized with Rab10 and Rab35 (Figure 2C). On the other hand, the signals of Rab8 and Rab32 (human Rab29 ortholog) in the Arl8-positive structures were comparable to the background signals (Figure 2C). Overexpression of Rab3 GDP-GTP exchange factor (Rab3-GEF), which activates Rab3 GTPase, suppressed Arl8 accumulation in *Lrrk*^*+/−*^, while knockdown of Rab3-GEF resulted in Arl8 accumulation (Figure 2D). GTPase inactive Rab3 T35N, a non-phosphorylated form of Rab3 T85A, and Rab32 WT also caused Arl8 accumulation (Figure 2E). However, Rab32 Q79L failed to increase Rab3 phosphorylation, which does not support the idea that Rab32 is an upstream regulator of Lrrk (Figure S2A). Unlike Rab3, overexpression of Rab8, Rab10, and Rab35 did not affect Arl8 accumulation (Figure 2E). Manipulation of Rab10 activity also did not affect the Arl8 phenotype (Figure S2B). These observations indicate that the inactivation of Rab3 GTPase, which is likely to be caused by loss of Lrrk, leads to Arl8 accumulation. Interestingly, the ectopic expression of Rab3 T35N and T85A was enriched in the distal axons (Figure S2C), while Rab3 Q80L and T85A were enriched in the proximal axons (arrows in Figure S2C). Rab3 Q80L and T35N were also enriched in cell bodies (CB in Figure S2C), suggesting that the GTPase activity of Rab3 regulates the neuronal distribution of Rab3.

The reduction in Arl8 GTPase activity itself also affected the presynaptic accumulation of Arl8 because the knockdown of Arl8-GEF (BORCS5) suppressed its accumulation (Figure 2F). Overexpression of Arl8 WT promoted Arl8 accumulation without affecting bouton size, while overexpression of both Arl8 Q75L and T34N attenuated Arl8 accumulation (Figure 2G). Arl8 signals were distributed over the entire length of the axons in Arl8 Q75L-expressing flies and were enriched in the proximal axons of Arl8 T34N-expressing flies (Figure S2D). These results suggest that the GTPase activation cycle of Arl8 is required for its terminal accumulation.

### Transport turnaround is impaired by leucine-rich repeat kinase loss

**Figure 3:**
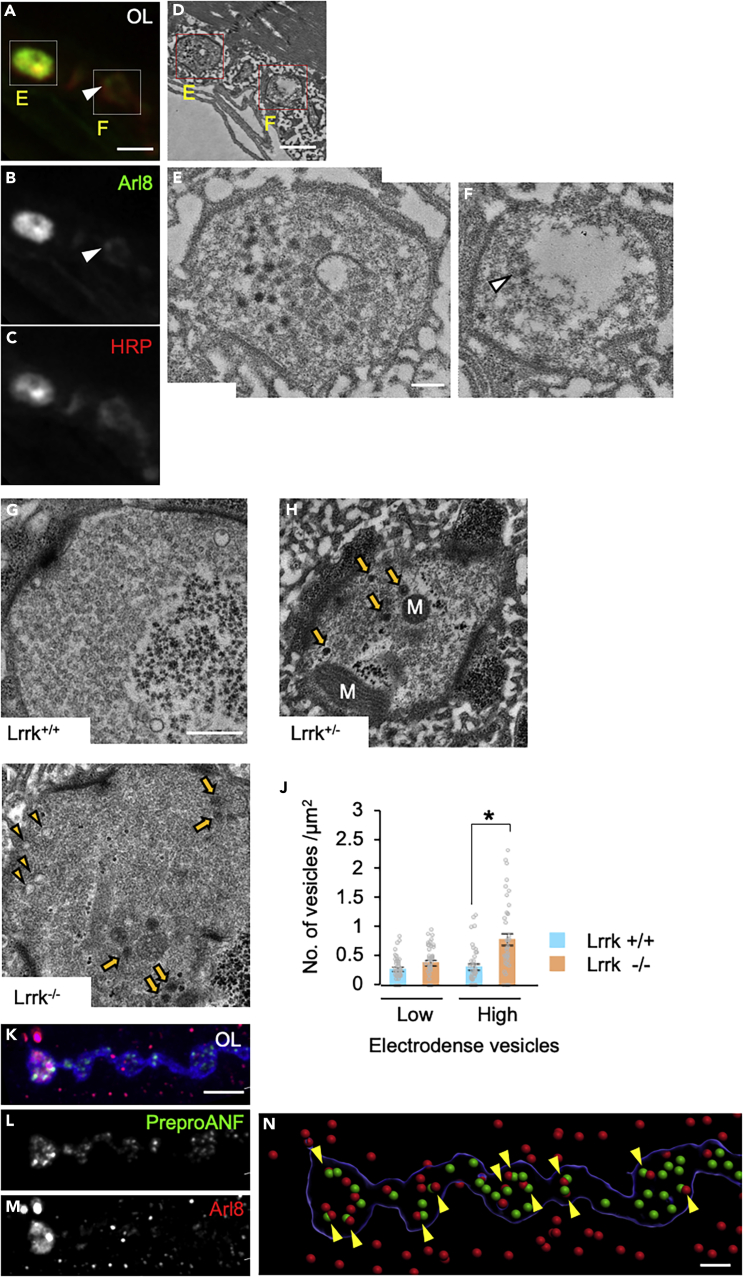
Loss of Lrrk leads to presynaptic DCV accumulation along with Arl8 (A–F) Arl8 aggregation-positive boutons exhibit an abundance of DCVs. Fluorescence images of Arl8 aggregation (green)-positive boutons (red). (D–F) The same boutons in A–C were analyzed by correlative light and electron microscopy (CLEM). A and D contain the most distal and second most distal boutons (corresponding to panels E and F). Scale bars, 20 μm (A–C); 1 μm (D); 200 nm (E, F). Arrowheads in A and B indicate the Arl8 enriched region of the second most distal bouton, which also contains DCV accumulation (F). (G–I) Ultrastructure of the synapses at the NMJ of the indicated genotypes. Arrowheads and arrows indicate low electrodense vesicles and high electrodense vesicles, respectively. M, mitochondrion. Scale bar, 500 nm. (J) Number of low and high electrodense vesicles. Graphs represent mean ± SEM (n = 31–46 synaptic boutons in 3–8 larvae). ∗p < 0.05, two-tailed *t-*test. (K–M) Distribution of PreproANF and Arl8 in *Lrrk*^*+/−*^ boutons. Scale bar, 5 μm. (N) Spot detection tool (Imaris, Oxford Instruments, 9.5.0) reveals co-localization (arrowheads) of PreproANF (green) and Arl8 (red). Note that Arl8 signals outside the synapses indicate the presence of lysosome-resident Arl8 in the muscles. Scale bar, 2 μm. See also Figure S3.

To clarify the type of organelle that exists in the synaptic boutons where Arl8 accumulates, we observed the ultrastructure of Arl8-positive boutons by correlative light and electron microscopy (Figures 3A-3F). In Arl8-positive boutons, many vesicular structures with high electron density were accumulated (Figures 3E and 3F). A detailed analysis with conventional electron microscopy revealed that *Lrrk*^*+/−*^ terminal boutons contained densely packed DCV-like structures (Figures 3G-3J). Moreover, Arl8 co-localized with PreproANF, a DCV marker, suggesting that Arl8 is involved in the axonal transport of DCVs (Figures 3K-3N). Chloroquine treatment, which causes de-acidification of acidic organelles such as DCVs and lysosomes,^48^ induced Arl8 accumulation and increased the number of DCVs at *Lrrk*^*+/+*^ presynaptic boutons, again suggesting that Arl8 is involved in DCV function (Figure S3).

**Figure 4:**
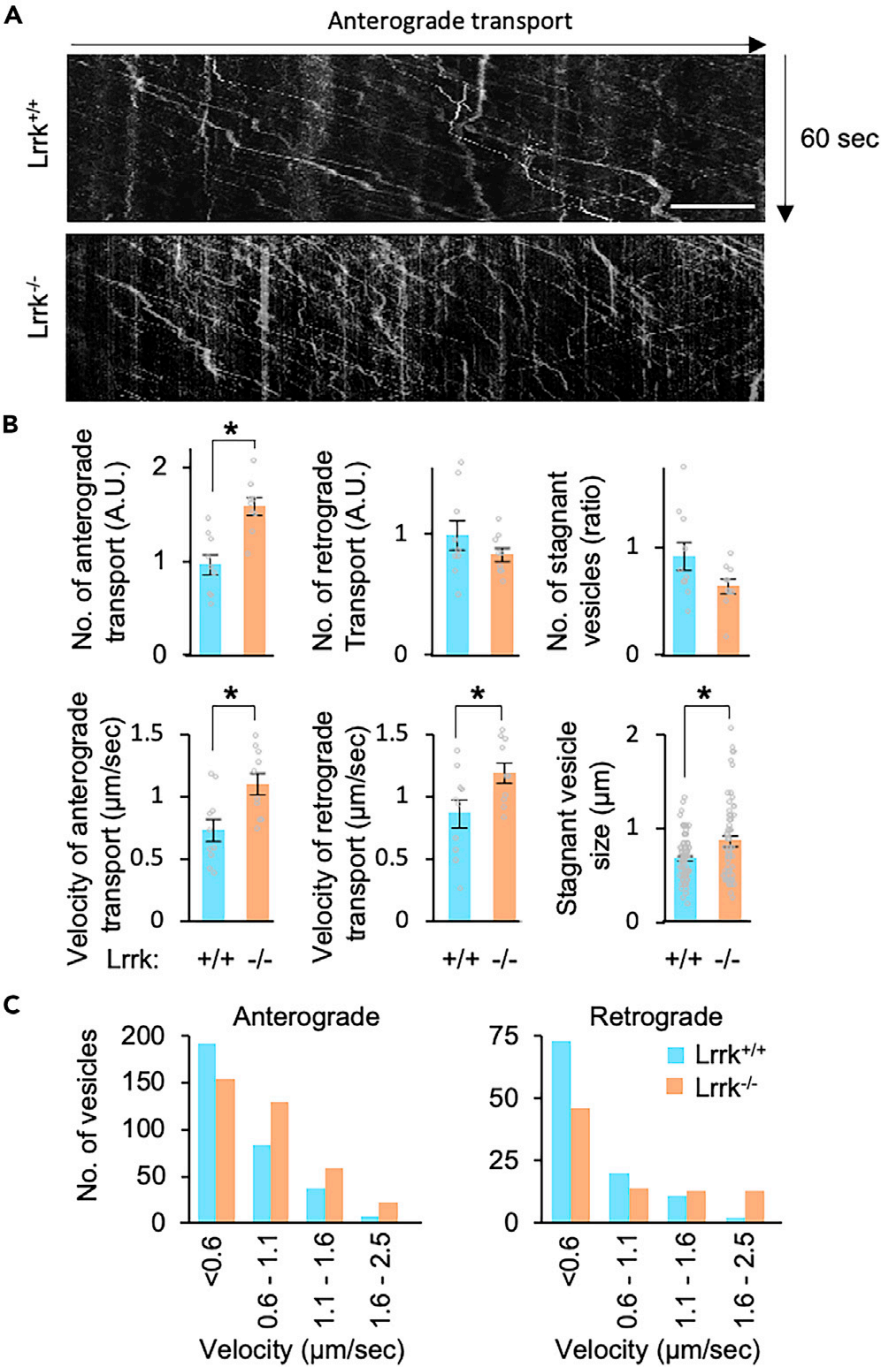
Lrrk regulates axonal transport of Arl8-positive vesicles (A) Representative kymographs of Arl8 axonal transport. Scale bar, 10 μm. (B) Velocity of Arl8 axonal transport, number of Arl8 signals, and size and number of stagnant Arl8 vesicles. Graphs represent mean ± SEM (n = 10 axons in 4 larvae). ∗p < 0.05, two-tailed *t-*test. (C) Distribution of the velocity of Arl8-positive vesicles in the presence or absence of Lrrk.

Trafficking of Arl8-positive puncta demonstrated that the vesicle number in anterograde transport increased in *Lrrk*^*−/−*^, while the vesicle number in retrograde transport was unchanged (Figures 4A and 4B). The velocity of transport in *Lrrk*^*−/−*^ was faster in both directions (Figures 4B and 4C). The size of stagnant puncta increased while their number remained unchanged (Figure 4B). These observations suggest that the accumulation of Arl8 by Lrrk loss is mainly due to the failure of the Arl8-positive DCVs to turn over at the presynaptic terminals.

### The anterograde motor Unc-104 and SYD/JIP4 are involved in Arl8 dynamics

**Figure 5:**
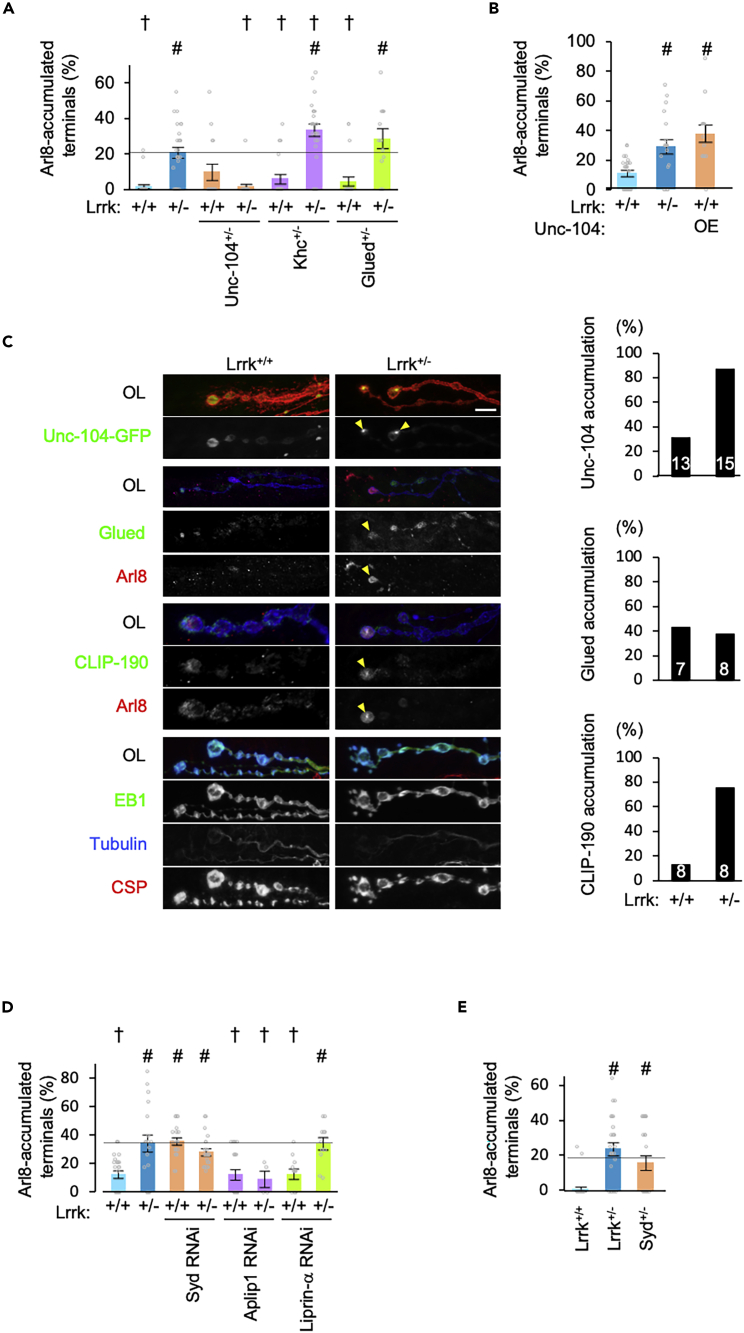
Arl8 transport is regulated by Unc-104 along with Lrrk (A) Reduction in Unc-104 rescues Arl8 accumulation through Lrrk loss. Arl8 accumulation ratio (as percentage) of the indicated genotypes on the *w*^*1118*^ genetic background are graphed (mean ± SEM, n = 12–26 NMJs in 4–10 flies). #p < 0.05 vs. Lrrk^+/+^; †p < 0.05 vs. Lrrk^+/−^ by Dunnett’s test. (B) Ectopic expression of Unc-104 promotes Arl8 accumulation. Flies with the indicated genotypes were analyzed on an *elav-GAL4* genetic background. Graphs represent mean ± SEM (n = 14–26 NMJs in 4–10 flies). #p < 0.05 vs. Lrrk^+/+^ by Dunnett’s test. (C) Unc-104 and CLIP-190 are accumulated in the Alr8 aggregates. Distribution of Unc-104, Glued, CLIP-190, or EB1 in *Lrrk*^*+/+*^ or *Lrrk*^*+/−*^ NMJs. Scale bar, 10 μm. Percentage of boutons with condensed co-localization of Unc-104, Glued, or CLIP-190 with Alr8 aggregates are graphed. n = 7–15 NMJs (indicated in graph) in 4–6 flies. (D and E) Reduction of Syd rescues Arl8 accumulation by Lrrk loss. Flies with the indicated genotypes were analyzed on an *elav-GAL4* (D) or *w*^*1118*^ (E) genetic background. Graphs represent mean ± SEM (n = 4–37 NMJs in 3–12 flies). #p < 0.05 vs. Lrrk^+/+^; †p < 0.05 vs. Lrrk^+/−^ by Dunnett’s test.

In *C*. *elegans*, the anterograde motor Unc-104/KIF1A is involved in the microtubule-dependent transport of Arl8-positive vesicles.^46^ Removal of a copy of the *Unc-104/KIF1A* gene, but not *Khc/KIF5*, suppressed the Arl8 accumulation in *Lrrk*^*+/−*^ (Figure 5A), while overexpression of *Unc-104* in the *Lrrk*^*+/+*^ genetic background resulted in Arl8 accumulation (Figure 5B). On the other hand, the removal of one copy of the retrograde motor *Glued/Dynactin* had no effect on the Arl8 phenotype in either *Lrrk*^*+/+*^ or *Lrrk*^*+/−*^ (Figure 5A). A mammalian ortholog of *Drosophila* CLIP-190, CLIP170, is involved in the initiation of Dynein-dependent retrograde transport at the microtubule plus-end.^49^ Unc-104 and a microtubule-plus-end-tracking protein (+TIP), CLIP-190, were also accumulated at the Arl8 accumulation site, while other + TIPs EB1 and Glued were not (Figure 5C). Knockdown or removal of one copy of *SYD/JIP4*, which reportedly regulates the sorting of SV proteins along with *lrk-1* at the *trans-*Golgi network (TGN) in *C*. *elegans*, recapitulated the Arl8 accumulation in *Lrrk*^*+/+*^ (Figures 5D and 5E).^41^ In combination of *SYD/JIP4* RNAi with *Lrrk*^+/−^, there were no additive effects on Arl8 accumulation (Figure 5D). In contrast, inhibition of *Aplip1/JIP1* in *Lrrk*^*+/−*^ rescued Arl8 accumulation (Figure 5D). Knockdown of the DCV-tethering factor Liprin-α did not affect Arl8 accumulation (Figure 5D).^50^ These results indicate that the dysregulation of Unc-104 and SYD is involved in Arl8-positive DCV accumulation.

### Presynaptic phenotypes by loss of Parkinson’s disease-causative/risk genes that regulate membrane dynamics

**Figure 6:**
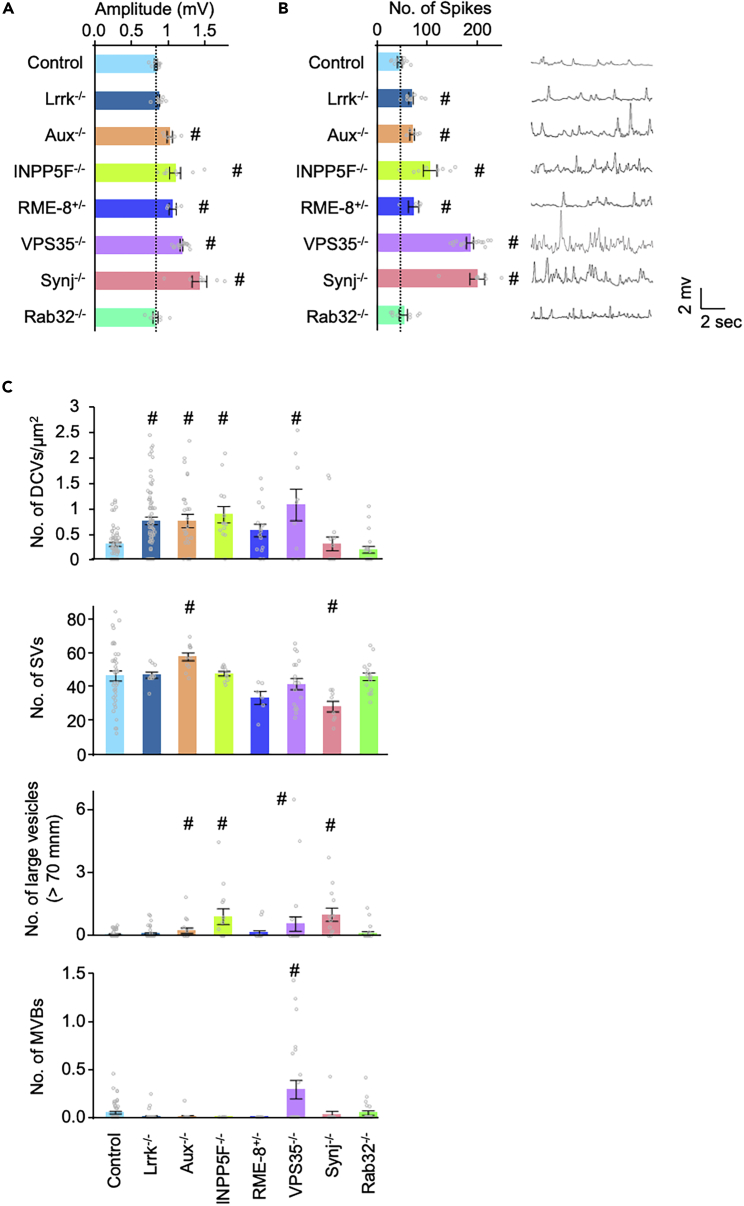
Electrophysiological and morphological phenotypes of LOF mutants of PD-causative/risk genes (A and B) Averaged amplitudes (A), frequency (left in B) of mEJP for 30 s, and representative mEJP traces (right in B) in larval NMJ. (C) The number of DCVs, SVs, large vesicles (>70 nm in diameter), and MVBs in the unit area (500-nm square area containing T bar) of the NMJ. #p < 0.05 vs. normal control (*w*^*1118*^) by Dunnett’s test. See also Figure S4.

Loss of LRRK2 or VPS35 causes defects in endocytosis and SV recycling at synapses, resulting in abnormal presynaptic activity.^35^ To determine whether this is a common phenotype for PD-associated genes that are believed to be involved in vesicle trafficking,^51^ we performed electrophysiological analysis and ultrastructural observation of synaptic boutons using flies harboring LOF alleles for *Rab32*, *Lrrk*, *VPS35*, *Aux*, *INPP5F*, *Synaptojanin* (*Synj)*, and *RME-8* (Figure 6). Among them, we employed heterozygous *RME-8* LOF flies because homozygous LOF files were lethal at a very early developmental stage. The amplitude (Figure 6A) of miniature excitatory junction potentials (mEJPs) showing spontaneous firing was increased in most mutant flies except *Lrrk*^*−/−*^ and *Rab32*^*−/−*^, compared to normal control. The frequency of mEJP was also increased in all mutant flies except *Rab32*^*−/−*^ (Figure 6B).

Ultrastructural observations of synaptic boutons revealed that the DCV density was increased in *Lrrk*^*−/−*^, *Aux*^*−/−*^, *INPP5F*^*−/-*^, and *VPS35*^*−/−*^, while the SV number around the active zones was reduced and increased in *Synj*^*−/−*^ and *Aux*^*−/−*^, respectively (Figures 6C and S4). Large vacuoles were frequently observed in *Aux*^*−/−*^, *INPP5F*^*−/-*^, *VPS35*^*−/−*^, and *Synj*^*−/−*^, and intraluminal vesicles, typical of multivesicular body (MVB), were frequently observed in *VPS35*^*−/−*^ (Figures 6C and S4). These results imply that mutations in PD-associated genes other than *Rab32* impair synaptic bouton function and that increased DCV density is a common phenotype in most of the PD-associated genes we analyzed.

### Parkinson’s disease-causative/risk genes, VPS35, RME-8, INPP5F, and auxilin modulate the Arl8 phenotype

**Figure 7:**
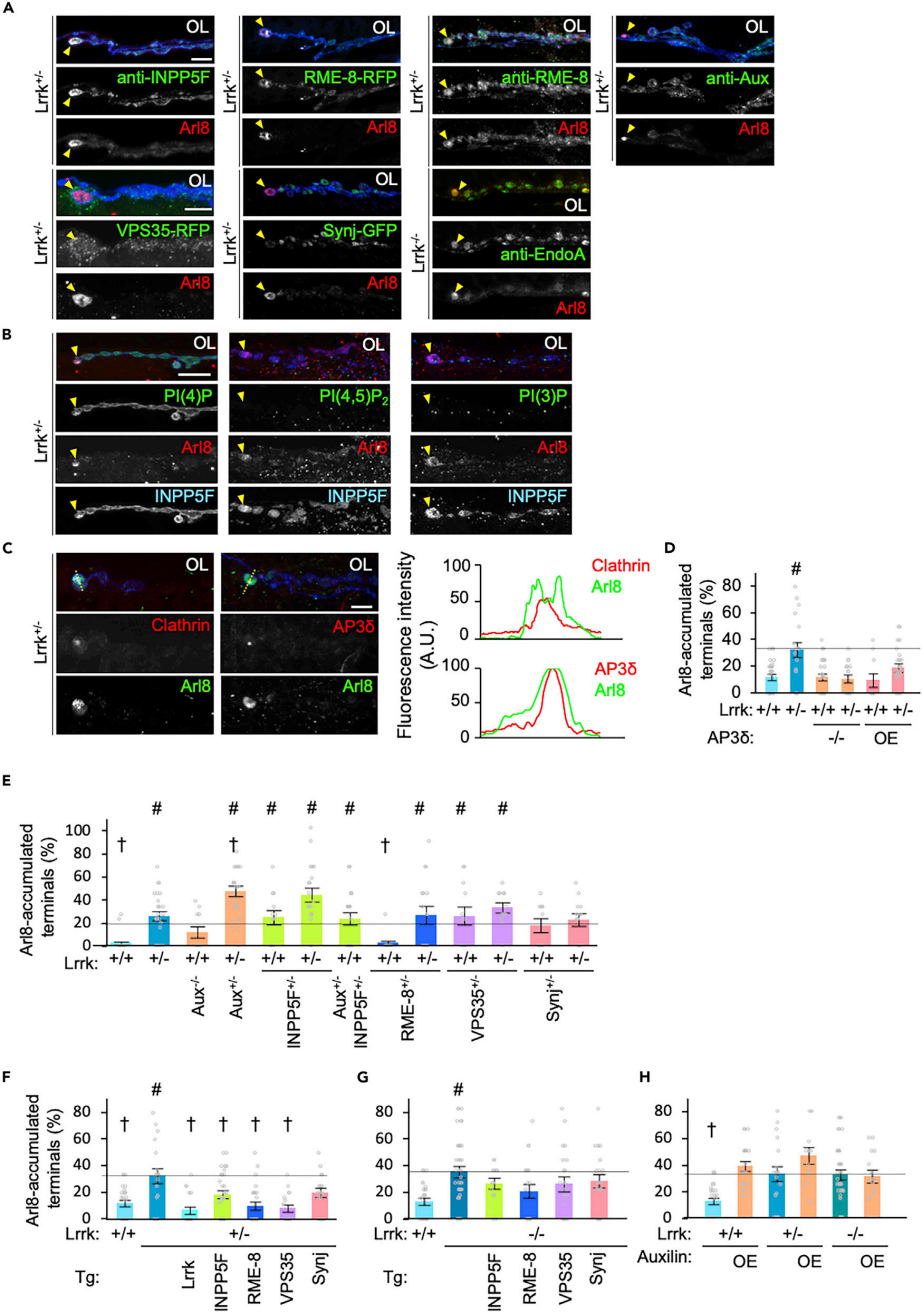
Genetic manipulation of Aux, RME-8, VPS35, and INPP5F affects Arl8 accumulation (A) Distribution of PD-causative/risk genes in *Lrrk*^*+/−*^ or *Lrrk*^*−/−*^ NMJs. RME-8-RFP, VPS35-RFP, and Synj-GFP were expressed using *elav-GAL4* and other PD-causative/risk genes were stained using specific antibodies. Synaptic boutons and Arl8 in the NMJ were visualized with anti-Arl8 (red) and DyLight649-conjugated anti-HRP (blue), respectively. Arrowheads indicate Arl8 aggregates. Scale bar, 10 μm. EndoA, Endophilin A. (B) PI(4)P, but not PI(4,5)P_2_ or PI(3)P, is enriched in Arl8 aggregates (arrowheads). Scale bar, 10 μm. (C) Clathrin and AP3δ are co-localized in Arl8 aggregates. Line profiles suggest that clathrin and AP3δ were localized in the core of Arl8 aggregates. Scale bar, 5 μm. (D) Altered expression of AP3δ affects Arl8 aggregates. Graphs represent mean ± SEM (n = 10–26 NMJs in 4–7 flies on an *elav-GAL4* genetic background). #p < 0.05 vs. normal control (*w*^*1118*^) by Dunnett’s test. (E) Loss of Aux and INPP5F promotes Arl8 aggregation. Graphs represent mean ± SEM (n = 10–37 NMJs in 4–10 flies on a *w*^*1118*^ genetic background). #p < 0.05 vs. Lrrk^+/+^; †p < 0.05 vs. Lrrk^+/−^ by Dunnett’s test. (F, G) Ectopic expression of INPP5F, RME-8, or VPS35 suppresses Arl8 aggregation in *Lrrk*^*+/−*^ (F), but not in *Lrrk*^*−/−*^ (G). Graphs represent mean ± SEM (n = 11–36 NMJs in 4–10 flies on an *elav-GAL4* genetic background). #p < 0.05 vs. Lrrk^+/+^; †p < 0.05 vs. Lrrk^+/−^ by Dunnett’s test. (H) Ectopic expression of Aux itself promotes Arl8 aggregation. Graph represents mean ± SEM (n = 11–36 NMJs in 4–10 flies on an *elav-GAL4* genetic background). †p < 0.05 vs. Lrrk^+/−^ without Aux OE by Dunnett’s test. See also Figures S5 and S6.

Next, we searched for PD-associated genes that co-localize with Arl8 accumulation among the above genes. Synaptic endocytosis-related molecules, VPS35, Synj, and Endophilin A, which are PD-associated proteins,^52^^,^^53^^,^^54^^,^^55^^,^^56^^,^^57^^,^^58^ were partly or not concentratedly localized with Arl8 accumulation (Figure 7A). In contrast, PD-associated RME-8 and INPP5F accumulated in Arl8 aggregates, in which phosphatidylinositol 4-phosphate [PI(4)P] was also enriched (Figures 7A, 7B, and S5A–S5D).^9^^,^^59^^,^^60^ On the other hand, PI(4,5)P_2_ and PI(3)P were not enriched in Ar8 aggregates (Figure 7B). Overexpressed Aux also accumulated in Arl8 aggregates, while endogenous Aux was partly co-localized (Figures 7A and S5E–S5G). Furthermore, clathrin and the component of the AP3 complex at the TGN, AP3δ, co-localized with Arl8, suggesting that molecules involved in the clathrin-mediated vesicle formation from the TGN were also ectopically accumulated (Figures 7C, S5H, and S5I).^61^^,^^62^^,^^63^ In contrast, both the overexpression and the removal of AP3δ suppressed Arl8 accumulation in Lrrk^+/−^ (Figure 7D). To further elucidate the genetic involvement of the above PD-associated genes, we first examined whether Arl8 accumulation could be reproduced by the LOF alleles of each gene. The introduction of a single LOF allele of INPP5F and VPS35 caused Arl8 accumulation similar to that caused by Lrrk mutations (Figure 7E). When we combined *Lrrk*^*+/−*^ with the LOF allele of each gene, Arl8 accumulation was aggravated by the combination of the Aux LOF allele and tended to be enhanced by the INPP5F LOF allele (Figure 7E). On the other hand, the overexpression of INPP5F, RME-8, and VPS35 reduced the Arl8 accumulation in *Lrrk*^*+/−*^ flies (Figure 7F). However, homozygous loss of *Lrrk* abolished these rescue effects (Figure 7G). These results suggest that INPP5F, RME-8, and VPS35 are genetically upstream of Lrrk. Overexpression of Aux resulted in Arl8 accumulation regardless of Lrrk activity (Figure 7H). The results of *Aux* and *AP3δ* may suggest that *Aux* and *AP3* are downstream of Lrrk. The results of these genetic tests are summarized in Figure S6.

### α-Synuclein is incorporated into Arl8 aggregates

**Figure 8:**
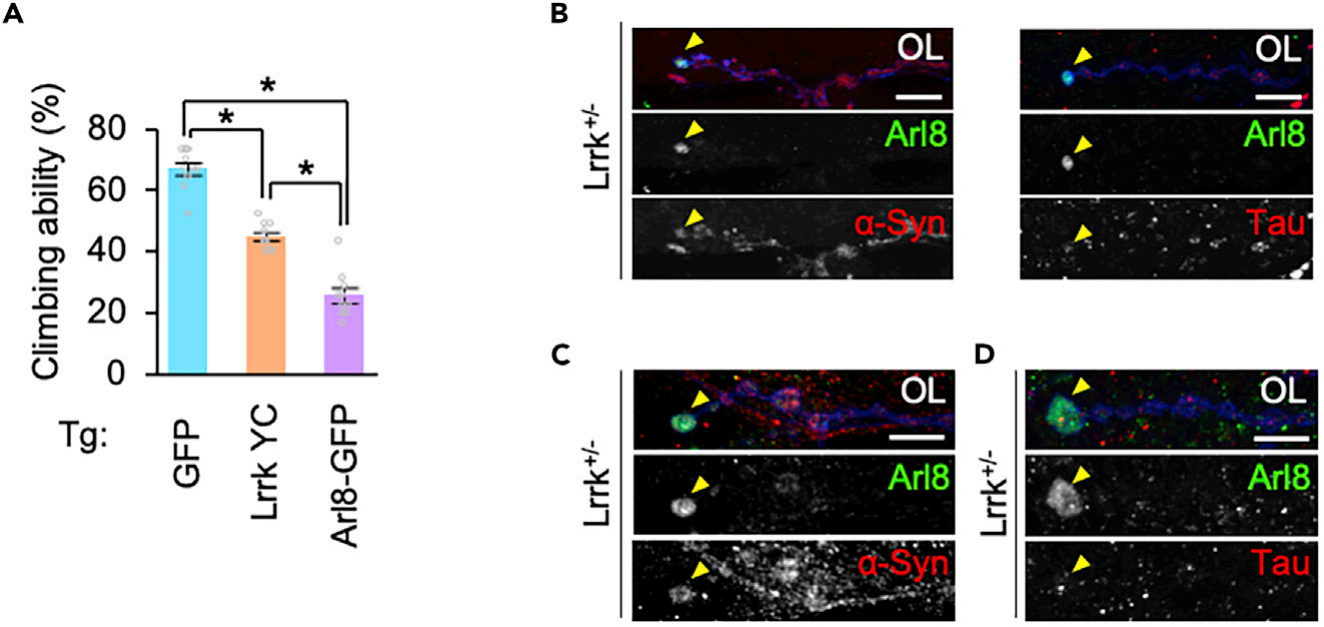
Synaptic activity-dependent co-localization of α-Synuclein with Arl8 aggregates (A) Ectopic expression of Arl8-GFP or Lrrk Y1383C in dopaminergic neurons impairs motor ability. A climbing assay was performed to estimate the motor function. GFP served a control. n = 10 trials with 50 flies aged 14 days old, ∗p < 0.0001 by Tukey–Kramer test. (B) Presynaptic localization of α-Synuclein and Tau in *Lrrk*^*+/−*^ NMJs. α-Synuclein (left) and Tau (right) neuronally expressed by *elav-GAL4* were stained with specific antibodies. Synaptic boutons in the NMJ were visualized with DyLight649-conjugated anti-HRP (blue). Arrowheads indicate Arl8 aggregates. (C, D) External α-Synuclein (C) and Tau (D) are incorporated into presynaptic Arl8 aggregates after synaptic stimulation. The NMJs of *Lrrk*^*+/−*^ flies incubated with recombinant α-Synuclein or Tau were electrostimulated (5 V, 1 Hz) for 10 min and visualized with specific antibodies. Scale bars, 10 μm in B-D. See also Figure S7.

The consequence of Arl8 accumulation at the presynapses was first estimated by a behavioral assay. Overexpression of Arl8 in dopaminergic neurons of adult flies impaired motor ability, suggesting that the constitutive accumulation of Arl8 at the presynapses is neurotoxic (Figure 8A). The pathology of PD linked to LRRK2 mutations is variable, with the accumulation of α-Synuclein, tau, or Aß.^20^^,^^64^ This pleiomorphic pathology suggests that mutations in LRRK2 result in an aggregation-prone brain environment rather than direct involvement in the aggregation of these molecules. The aggregation and propagation of α-Synuclein and Tau is an important aspect of aging-dependent neurodegenerative etiology, including that of PD where it is unclear how aggregation forms and propagates through neural circuits. Since the accumulation of Arl8 is observed not only in flies expressing PD-related *Lrrk* mutants but also in *Lrrk* knockout flies, we used *Lrrk*^*+/−*^ flies to test whether Arl8 accumulations could be a location of α-Synuclein and Tau aggregation. α-Synuclein and Tau expressed ectopically in larval motor neurons were not enriched in the Arl8 accumulation of *Lrrk*^*+/−*^ flies (Figure 8B). However, α-Synuclein incorporated from the synaptic cleft by synaptic activity-dependent uptake was accumulated in the Arl8-positive structures (Figure 8C). In contrast, Tau incorporated under the same conditions did not accumulate (Figure 8D). Next, we examined whether Arl8 is present in Lewy bodies, the neuronal inclusions of α-Synuclein in humans. Lewy bodies-positive dopaminergic neurons accounted for 14.97 ± 2.90% of total midbrain dopaminergic neurons (n = 56.83 ± 10.55) in patients with PD we analyzed (Figure S7 and Table S1). Dopaminergic neurons with Arl8B-positive Lewy bodies, in which Arl8B-immunosignals were positive in their marginal part, constituted 40.87 ± 10.42% and no Arl8A-positive Lewy bodies were detected (Figure S7 and Table S1).

## Discussion

Neurophysiological and morphological analyses of our and other studies have suggested that Lrrk is involved in presynaptic functions in *Drosophila*.^35^^,^^43^^,^^44^ The present study reports that the dysregulation of Lrrk activity resulted in presynaptic Arl8 accumulation, possibly due to DCV accumulation. Further, Rab3 co-localized with Arl8 accumulation. Moreover, the expression of a dominant-negative Rab3 mutant and a putative phospho-resistant Rab3 mutant by Lrrk in neurons recapitulated Arl8 accumulation, implying that defects in DCV exocytosis contribute to Arl8 accumulation. Arl8 was transported to the axonal terminals via Unc-104-dependent anterograde transport. The presynaptic Arl8 accumulation may involve a switching defect in the retrograde transport at the microtubule plus-end. This is because Lrrk loss increased the number of Arl8-positive vesicles transported in the anterograde direction, but not in the retrograde direction (Figure 4B). The enriched co-localization of CLIP-190 with Arl8 at the microtubule plus-ends also indicates this notion (Figure 5C). In mammalian neurons, PD-associated LRRK2 G2019S has been implicated in facilitating anterograde transport of autophagic vesicles that should be retrograded by dynein.^34^ The enhanced anterograde transport by LRRK2 G2019S involves the recruitment of JIP4 into autophagic vesicles via increased phosphorylation of Rab10 by LRRK2 G2019S and activation of kinesin.^34^ In contrast, a LRRK2 kinase inhibitor inhibits the anterograde transport of autophagic vesicles.^34^ Hence, the molecular mechanism observed in this context does not appear to be directly associated with the mechanism of Arl8 accumulation by Lrrk loss or the PD-associated Lrrk Y1383C mutant. Phosphorylation of CLIP170 by LRRK1 enhances the binding of CLIP170 to the Dynein motor.^65^ Because *Drosophila* Lrrk is also an ortholog of LRRK1, the recruitment activity of the Dynein motor to the plus-end of microtubules may be reduced in *Lrrk*^*+/−*^. However, the observation that Arl8 also accumulates in the Lrrk Y1383C mutant with enhanced kinase activity suggests the existence of other mechanisms (Figure 1C).^42^

Arl8 is a marker for lysosomes in the cell body and a marker for presynaptic lysosome-related vesicles (PLVs) in the presynapses.^45^ Both lysosomes and PLVs share the common feature of being acidic organelles. Arl8 is believed to be involved in their transport. Further, PLVs may contain DCVs; several lines of evidence support this idea: First, CLEM revealed an increased distribution density of DCVs, but not SVs, in synaptic boutons containing Arl8 accumulation. Second, DCV aggregation is observed in the *lrk-1* mutant in *C*. *elegans*^66^; an increase in the size of Arl8-positive stagnant vesicles is also observed in this study (Figure 4B). Third, Rab3 has been reported to be involved in DCV exocytosis, but not SV.^67^ Fourth, the AP-3 complex contributes to the formation and maturation of DCV at the TGN.^63^^,^^68^ The above reports indicate that Lrrk and AP-3 dysfunction caused the missorting of DCV proteins such as synaptotagmin, leading to the generation of incompetent DCVs.^63^ Incompetent DCVs could result in inadequate neuroaxonal transport or the incomplete docking of DCVs to the membrane and subsequent exocytosis, leading to the accumulation of DCVs and Arl8 in the distal boutons. The change in the velocity of Arl8 axonal transport by Lrrk loss may be due to an event such as a wrong motor adapter being loaded on the DCV or the Arl8 or its adaptors being loaded on different vesicles. These possibilities may explain our observations that are difficult to interpret, such as enhanced Arl8 accumulation in *Lrrk*^*+/−*^ by *Khc*^+/−^ (Figure 5A) and suppression of the Arl8 accumulation by *Aplip1/JIP1* RNAi (Figure 5D), despite the involvement of both UNC-104 and Khc motors in DCV transport in different regions of neurons.^69^

The phenotype of Arl8 accumulation in the terminal boutons allowed us to assess the genetic interaction of Lrrk with known PD-related genes (Figure S6). LOF mutations of Aux and INPP5F and overexpression of Aux and Rab32 enhanced Arl8 accumulation by *Lrrk*^*+/−*^. In contrast, overexpression of RME-8, INPP5F, and VPS35 suppressed Arl8 accumulation by *Lrrk*^*+/−*^, while this effect disappeared in the *Lrrk*^*−/−*^ background, suggesting that RME-8, INPP5F, and VPS35 are genetically upstream of Lrrk. The genetic interaction of VPS35 with Lrrk is supported by our previous study and others^14^^,^^35^^,^^70^; RME-8, together with a retromer containing VPS35, is involved in microtubule-dependent tubulation of vesicles.^1^ On the other hand, LRRK2 also regulates the microtubule-dependent tubulation of lysosomes via JIP4.^33^ These observations suggest that RME-8 along with LRRK2 may regulate the microtubule-dependent membrane dynamics of DCVs. INPP5F is a PI4P phosphatase^10^ and is suggested to function with activated LRRK2 in ruptured lysosomes.^33^ INPP5F is also involved in insulin granule exocytosis, and recruitment of INPP5F to the insulin granules requires active Rab3 and PI4P.^13^ Arl8-accumulated vesicles were PI4P- and Rab3-positive and INPP5F was also co-localized with Arl8-accumulated vesicles, indicating that Lrrk may be involved in the dephosphorylation of PI4P by INPP5F through Rab3 activation (Figures 2C, 7A and 7B).

Similar to Lrrk, both overexpression and LOF mutations of Aux enhanced Arl8 accumulation. Aux may require appropriate regulation in terms of clathrin uncoating activity. Clathrin and AP3δ also co-localize in the core of Arl8 aggregates, suggesting the missorting of clathrin-dependent budding vesicles from the TGN (Figure 7C). In a previous study, LRRK2 was reported to regulate endocytosis of clathrin-coated vesicles via phosphorylation of the μ2 subunit of the AP-2 complex (AP2M1). Both decreased phosphorylation of AP2M1 by LRRK2 loss and increased phosphorylation of AP2M1 by pathogenic LRRK2 G2019S impair endocytosis of clathrin-coated vesicles and affect dopaminergic neuron viability.^71^

Interestingly, LRK-1 and UNC-16/JIP4 have been asserted to be required for UNC-104-dependent axonal transport of the SV proteins Rab3 and SNB-1 in *C*. *elegans*, in which the AP-3 complex appears to have a role in SV biogenesis downstream of LRK-1 and UNC-16 ^40,41^. In a different context in *C*. *elegans*, where ectopic extension of axonal terminals was observed due to *LRK-1* loss, the AP-3 complex is also implied to be genetically downstream of *LRK-1*.^16^ This study suggests that *glo-1*, which is an ortholog of mammalian Rab29/Rab7L, Rab32, and Rab38, is an upstream regulator of *LRK-1*.^16^ In *Drosophila*, the only *Rab32* gene is an ortholog of mammalian Rab29 and Rab32. However, the overexpression of Rab32 Q79L failed to stimulate Rab3 phosphorylation, which argues against the possibility that Rab32 is an upstream regulator of Lrrk (Figure S2A). These genetic studies at least suggest that Aux, RME-8, VPS35, and INPP5F may be involved in the LRRK2 pathway, but details at the molecular level remain to be elucidated.

Although the involvement of Arl8 accumulation in PD pathogenesis is not clear, overexpression of Arl8 in dopaminergic neurons impaired motor ability (Figure 8A). Intriguingly, α-Synuclein incorporated in a neuronal activity-dependent manner was enriched in Arl8 aggregation (Figure 8C). Moreover, Arl8b immunosignals were partially present in Lewy bodies in dopaminergic cell bodies of patients with PD, suggesting that Arl8 accumulation at presynapses could be one of the locations of α-Synuclein aggregation. Alternatively, Arl8b may have been incorporated into α-Synuclein fibrils with lysosomes and/or related organelles during the formation of inclusion bodies (Figure S7 and Table S1).^72^^,^^73^

Roles of LRRK2 in autophagy and lysosomes have been described in mammals.^74^ In *Drosophila*, Lrrk is suggested to be involved in EndoA-mediated autophagy at the presynapses^75^ and in lysosomal positioning.^76^ The current study does not reveal whether the presynaptic accumulation of Arl8 and DCVs is associated with the alteration of the autophagy-lysosome pathway. However, since Arl8 accumulation was partially co-localized with EndoA, it may impair EndoA function (Figure 7A). In addition, since chloroquine treatment promoted the Arl8 accumulation, there may be some relationship between impaired acidification of acidic organelles, such as DCVs and lysosomes, and Arl8 accumulation (Figure S3).

In conclusion, this study reports that *Drosophila* Lrrk mutations lead to Arl8 accumulation at presynapses, postulating that Arl8 accumulation is a consequence of DCV accumulation and dependent on UNC-104 activity. Moreover, we suggest that known PD-related genes, Aux, RME-8, VPS35, and INPP5F, may ensure the precise transport of DCV-related proteins at the presynapses in cooperation with Lrrk and possibly JIP4 and AP3. Stagnation and ectopic accumulation of proteins at the presynapse may pose a risk for the aggregation of various neurodegeneration-related molecules, including α-Synuclein. Perry syndrome, in which Dynactin mutations cause protein stagnation at the presynapses, leading to neurodegeneration, is one example.^77^ Further analysis is needed to determine whether logistic errors in axonal transport are one of the causes of PD pathomechanism.

### Limitations of the study

The molecular mechanism of Arl8 accumulation via dysregulation of Lrrk kinase activity has not been determined in this study. The dysregulation of Rab3 activity and missorting of Arl8 at the TGN by Lrrk mutations is one possibility, while loss of Lrrk also appears to activate UNC-104-dependent anterograde transport of Arl8. Moreover, the GTPase-active form of Alr8 itself has been reported to activate UNC-104.^78^ This seemingly complex molecular relationship needs to be clarified. Furthermore, whether the accumulation of DCVs is involved in the pathogenesis of PD has not been completely analyzed in this study. DCVs are not only vesicles in which dopaminergic neurons store dopamine but also acidic organelles that are similar in nature to lysosomes, which have recently attracted attention in PD research. The possible involvement of DCV dysregulation in this disease should also be investigated in the future.

## STAR★Methods

### Key resources table

**Table:** 

REAGENT or RESOURCE	SOURCE	IDENTIFIER
**Antibodies**		

Anti-*Drosophila* Arl8, rabbit	Developmental Studies Hybridoma Bank (DSHB)	Arl8, RRID: AB_2618258
Anti-*Drosophila* Rab7, mouse	DSHB	Rab7, RRID: AB_2722471
Anti-*Drosophila* Calnexin 99A, mouse	DSHB	cnx99A 6-2-1, RRID: AB_2722011
Anti-*Drosophila* Hrs, mouse	DSHB	Hrs 27-4, RRID: AB_2618261
Anti-*Drosophila* LAMP1, rabbit	Abcam	RRID: AB_775973
Anti-*Drosophila* Rbsn5, rabbit	Tanaka and Nakamura, 2008^82^	N/A
Anti-*Drosophila* Endophilin A, guinea pig	Verstreken et al., 2002^83^	GP69, N/A
Anti-Ref(2)P, rabbit	Ikeda et al., 2019^84^	N/A
Anti-*Drosophila* INPP5F, guinea pig	This paper	N/A
Anti-*Drosophila* LRRK, rabbit	Imai et al., 2008^42^	N/A
Anti-*Drosophila* Aux, guinea pig	This paper	N/A
Anti-*Drosophila* RME-8, rabbit	This paper	N/A
Anti-*Drosophila* Clathrin heavy chain, rabbit	This paper	N/A
Anti-α-Synuclein, rabbit	Abcam	MJFR1, ab138501, RRID: AB_2537217
anti-α-Synuclein, mouse	FUJIFILM-Wako	RRID: AB_516843
Anti-Tau (Tau-C, 424-438 aa), rabbit	Matsumoto et al., 2015^85^	N/A
Anti-α-Tubulin, mouse	Sigma-Aldrich	DM1A, RRID: AB_1904178
Anti-α-Tubulin, rabbit	Cell Signaling Technology	11H10, RRID: AB_2619646
Anti-Actin, mouse	Millipore	C4, RRID: AB_2223041
Anti-Arl8A, rabbit	Atlas	HPA038759, RRID: AB_2676193
anti-Arl8B, rabbit	Proteintech	13049-1-AP, RRID: AB_2059000
Anti-Tyrosine hydroxylase, chicken	Abcam	ab76442, RRID: AB_1524535
Anti-GAPDH, mouse	Proteintech	1E6D9, RRID: AB_2107436
Anti-pRab, rabbit	Abcam	MJFR20, RRID: AB_2814988
Anti-GFP	MBL	598, RRID: AB_591816
Anti-GFP-FITC, goat	Abcam	ab6662, RRID: AB_305635

**Experimental models: Organisms/strains**		

*Drosophila melanogaster UAS-LAMP1-GFP*; *n-Syb-GAL4*	Bloomington Drosophila Stock Center (BDSC)	RRID: BDSC _42714
*Drosophila melanogaster elav-GAL4*	BDSC	RRID: BDSC _8765
*Drosophila melanogaster D42-GAL4*	BDSC	RRID: BDSC _8816
*Drosophila melanogaster Da-GAL4*	BDSC	RRID: BDSC _55850
*Drosophila melanogaster Dpp-GAL4*	BDSC	RRID: BDSC _1553
*Drosophila melanogaster TI{TI}Rab3[EYFP]*	BDSC	RRID: BDSC _62541
*Drosophila melanogaster TI{TI}Rab8[EYFP]*	BDSC	RRID: BDSC _62546
*Drosophila melanogaster TI{TI}Rab10[EYFP]*	BDSC	RRID: BDSC _62548
*Drosophila melanogaster TI{TI}Rab32[EYFP]*	BDSC	RRID: BDSC _62558
*Drosophila melanogaster TI{TI}Rab35[EYFP]*	BDSC	RRID: BDSC _62559
*Drosophila melanogaster UAS-Rab3 GEF RNAi*	BDSC	RRID: BDSC _28954
*Drosophila melanogaster UAS-Rab3 GEF [SC225]*	BDSC	RRID: BDSC_78052
*Drosophila melanogaster UASp-EYFP-Rab3 WT*	BDSC	RRID: BDSC _9762
*Drosophila melanogaster UASp-EYFP-Rab3 T35N*	BDSC	RRID: BDSC _9766
*Drosophila melanogaster UASp-EYFP-Rab3 Q80L*	BDSC	RRID: BDSC _9764
*Drosophila melanogaster UASp-EYFP-Rab8 WT*	BDSC	RRID: BDSC _9782
*Drosophila melanogaster UASp-Rab10-YFP WT*	BDSC	RRID: BDSC _9789
*Drosophila melanogaster UASp-YFP-Rab32 Q79L*	BDSC	RRID: BDSC _9816
*Drosophila melanogaster UAS-preproANF-Emerald*	BDSC	RRID: BDSC _7001
*Drosophila melanogaster unc-104*^*p350*^	BDSC	RRID: BDSC _24630
*Drosophila melanogaster Khc*^*8*^	BDSC	RRID: BDSC _1607
*Drosophila melanogaster UAS-unc-104-GFP*	BDSC	RRID: BDSC _24787
*Drosophila melanogaster Gl*^*1*^	BDSC	RRID: BDSC _510
*Drosophila melanogaster UAS-DCTN-GFP*	BDSC	RRID: BDSC _29983
*Drosophila melanogaster UAS-EB1-GFP*	BDSC	RRID: BDSC _36861
*Drosophila melanogaster Syd*^*z4*^	BDSC	RRID: BDSC _32016
*Drosophila melanogaster Aplip1*^*EK4*^	BDSC	RRID: BDSC _24632
*Drosophila melanogaster Aux*^*727*^	BDSC	RRID: BDSC _25672
*Drosophila melanogaster RME-8*^*D3*^	BDSC	RRID: BDSC _5525
*Drosophila melanogaster Vps35*^*MH20*^	BDSC	RRID: BDSC _67202
*Drosophila melanogaster Synj*^*1*^	BDSC	RRID: BDSC _24883
*Drosophila melanogaster Synj*^*2*^	BDSC	RRID: BDSC _24884
*Drosophila melanogaster Rab32*^*1*^	BDSC	RRID: BDSC _338
*Drosophila melanogaster TagRFP-T Vps35*	BDSC	RRID: BDSC _66527
*Drosophila melanogaster UAS-GFP-myc-2xFYVE*	BDSC	RRID: BDSC _42712
*Drosophila melanogaster UAS-PLC delta-PH-EGFP*	BDSC	RRID: BDSC_39693
*Drosophila melanogaster INPP5F*^*MI01858*^	BDSC	RRID: BDSC_34453
*Drosophila melanogaster garnet*^*1*^	BDSC	RRID: BDSC_3958
*Drosophila melanogaster UAS-Syd RNAi*	Vienna Drosophila Resource Center (VDRC)^86^	VDRC _101459
*Drosophila melanogaster UAS-Aplip1 RNAi*	VDRC^87^	VDRC _109501
*Drosophila melanogaster UAS-Liprin-α RNAi*	VDRC^88^	VDRC _106588
*Drosophila melanogaster UAS-BORCS5 RNAi*	VDRC^89^	VDRC _31570
*Drosophila melanogaster UAS-RME-8 RNAi*	VDRC	VDRC _22671
*Drosophila melanogaster UAS-Aux RNAi*	VDRC^90^	VDRC _103426
*Drosophila melanogaster UAS-Chc RNAi*	VDRC	VDRC _24789
*Drosophila melanogaster UAS-Chc RNAi*	VDRC	VDRC _23666
*Drosophila melanogaster pBac{DsRed}*^*LL05626*^	Kyoto Stock Center	#141867
*Drosophila melanogaster UAS-Aux RNAi*	NIG-Fly	1107R-2
*Drosophila melanogaster Arl8-GFP*	This paper	N/A
*Drosophila melanogaster UAS-EYFP-Rab3 T85A*	This paper	N/A
*Drosophila melanogaster UAS-Arl8 WT*	This paper	N/A
*Drosophila melanogaster UAS-Arl8 Q75L*	This paper	N/A
*Drosophila melanogaster UAS-Arl8 T34N*	This paper	N/A
*Drosophila melanogaster UAS-SidX2-P4M-GFP*	This paper	N/A
*Drosophila melanogaster UAS-INPP5F*	This paper	N/A
*Drosophila melanogaster UAS-tau 2N4R*	This paper	N/A
*Drosophila melanogaster dLRRK*^*NS1*^	Dodson et al., 2012^76^	Flybase ID: FBal0319307
*Drosophila melanogaster dLRRK*^*e03680*^	Imai et al., 2008^42^	Flybase ID: FBti0048428
*Drosophila melanogaster UAS-Arl8*.*GFP*	Rosa-Ferreira et al., 2018^91^	Flybase ID: FBtp0147747
*Drosophila melanogaster UAS-dLRRK WT*	Imai et al., 2008^42^	Flybase ID: FBal0220786
*Drosophila melanogaster UAS-dLRRK 3KD*	Imai et al., 2008^42^	Flybase ID: FBtp0040856
*Drosophila melanogaster UAS-dLRRK Y1383C*	Imai et al., 2008^42^	Flybase ID: FBal0220787
*Drosophila melanogaster UAS-dLRRK I1915T*	Imai et al., 2008^42^	Flybase ID: FBtp0040855
*Drosophila melanogaster UAS-hLRRK I2020T*	Venderova et al., 2009^92^	Flybase ID: FBal0244982
*Drosophila melanogaster UAS-dVPS35 WT*	Inoshita et al., 2017^35^	N/A
*Drosophila melanogaster UAS-spin-myc-RFP*	Sweeney and Davis, 2002^93^	Flybase ID: FBtp0023043
*Drosophila melanogaster Rab10 null*	Kohrs et al., 2021^94^	Flybase ID: FBal0370495
*Drosophila melanogaster UAS-Crag*	Nakamura et al., 2020^95^	Flybase ID: FBtp0142307
*Drosophila melanogaster UAS-CLIP-190-GFP*	Beaven et al., 2015^96^	Flybase ID: FBtp0108854
*Drosophila melanogaster Aux*^*I670K*^	Zhou et al., 2011^5^	Flybase ID: FBal0215598
*Drosophila melanogaster UAS-RME-8-RFP*	Chang et al., 2004^97^	Flybase ID: FBal0189648
*Drosophila melanogaster UAS-Aux-GFP*	Zhou et al., 2011^5^	N/A
*Drosophila melanogaster UAS-Aux*	Hagedorn et al., 2006^98^	N/A
*Drosophila melanogaster UAS-Synj-GFP-HA*	Dickman et al., 2005^99^	Flybase ID: FBtp0022534
*Drosophila melanogaster UAS-α-Synuclein LP2*	Trinh et al., 2008^100^	N/A
*Drosophila melanogaster tub-mCherry-AP3δ*	Burgess et al., 2011^101^	Flybase ID: FBal0277298
*Drosophila melanogaster UAS-hGalT-TagRFP*	Zhou et al., 2014^102^	Flybase ID: FBst0065251
*Drosophila melanogaster Clc-GFP*	Chang et al., 2002^103^	Flybase ID: FBst0007107
*Drosophila melanogaster tagBFP-FLAG-Rab35*	Gift from Dr. A. Satoh	N/A

**Software and algorithms**		

ImageJ Fiji v1.0	http://fiji.sc	RRID:SCR_002285
JMP v11.0.0	SAS	RRID:SCR_014242
Zen	www.zeiss.com	ZEN Digital Imaging for Light Microscopy; RRID:SCR_013672
Mini Analysis Program	Synaptosoft	RRID:SCR_002184

### Resource availability

#### Lead contact

Further information and requests for resources and reagents should be directed to and will be fulfilled by the Lead Contact, Yuzuru Imai (yzimai@juntendo.ac.jp).

#### Materials availability

All unique/stable reagents generated in this study are available from the lead contact without restriction, except for some guinea pig antibodies, which are limited in quantity.

### Experimental model and subject details

#### *Drosophila* strains

Fly culture and crosses were performed using standard fly food containing yeast, cornmeal, and molasses, and the flies were raised at 25°C. The *w*^*1118*^ (*w*^*–*^) line was used as a wild-type genetic background. Complementary DNAs for *Arl8* and *INPP5F* were amplified by PCR from first-strand cDNA library of *w*^*1118*^ and subcloned into pUAST vector. Complementary DNA for 2N4R Tau was amplified by PCR from adult human brain first-strand cDNA library and subcloned into pUAST vector. GFP-P4M-SidMx2 (Addgene #78544) was subcloned into pUAST vector to visualize PI(4)P in flies. Complementary DNAs for *YFP-Rab3 T85A*, *Arl8 Q75L*, and *Arl8 T34N* were generated by PCR-based mutagenesis using *pUASp-YFP-Rab3* (Addgene #37687) or *UAS-Arl8* as templates. Transgenic lines were generated on a *w*^*1118*^ background (BestGene Inc., Chino Hills, CA, USA). Arl8-GFP knock-in line was generated by insertion of a liner sequence (GTSGGS) and EGFP just before the stop codon (WellGenetics Inc., Taipei, Taiwan). Information on individual fly strains and genotypes for experiments is listed in the key resources table and Table S2 and can be found on FlyBase (flybase.org) unless otherwise noted. Experiments and ethics regarding the use of genetically modified *Drosophila* were approved by the Juntendo University School of Medicine, Animal Experiment Committee (approval number: 24-14).

#### Clinical samples

Detailed clinical characteristics were obtained from a neurodegenerative disease database of the Department of Neurology at Juntendo University. Details on these patients are given in Table S1. All procedures performed in studies for brain autopsy were in accordance with the ethical standards of the Juntendo University School of Medicine Ethics Committee (approval number: 2019012) and with the 1964 Helsinki Declaration and its later amendments or comparable ethical standards. Neuropathological assessments for AD, PD, PDD, and DLB were conducted by pathologists from the Department of Neurology at Juntendo University. Written informed consent for autopsy and analysis of tissue sample data was obtained for all patients.

### Method details

#### Antibody production

Anti-Aux and anti-INPP5F polyclonal antibodies were raised against recombinant GST-tagged Aux (720–1165 aa) and GST-tagged INPP5F (744–1000 aa) produced in the *Escherichia coli* strain Rosetta 2 (Novagen, Merck, Darmstadt, Germany). Rabbit anti-RME-8 polyclonal antibody was raised against a mixture of synthetic peptides (C-ISTYNPDKLDLTNRWS-coNH2 and C-KDQRHDLFIADTTIRGY-coNH2) and purified through affinity chromatography (Japan Bio Services, Asaka, Japan). Rabbit anti-Chc polyclonal antibody was raised against a synthetic peptide (DDSTEHKNIIQMEPQLMC; Cosmo Bio, Tokyo, Japan).

The following primary antibodies were used for western blotting: anti-GFP (1:5000; 598, MBL, Tokyo, Japan), anti-pRab (1:1000; MJF-R20, Abcam, Cambridge, UK), anti-Lrrk (1:2000; in-house, 2136013), anti-Aux (1:1000; in-house, R1), anti-INPP5F (1:1000; in-house, GP-C2), anti-RME-8 (1:1000, in-house, 1738071), anti-Actin (1:10000; Millipore, MAB1501), anti-GAPDH (1:1000; Proteintech, 1E6D9), and anti-Tubulin (1:5000; Sigma-Aldrich, DM1A).

#### Western blot analysis

For phos-tag western blot analysis, 3 male fly heads were homogenized with 20 μL lysis buffer containing 50 mM Tris-HCl, pH7.6, 150 mM NaCl, 1% Triton X-100, and protease inhibitor cocktail (03969, Nacalai Tesque, Kyoto, Japan) using a motor-driven pestle. After the addition of 1 mM MnCl₂ and 40 units of Lambda protein phosphatase (P0753L, New England BioLabs, Ipswich, MA), the lysate was incubated at 30°C for 30 min and subjected to 10% gel containing 50 μM Phos-tag Acrylamide (304–93521, FUJIFILM Wako, Osaka, Japan). For protein expression analysis, the fly brain and thorax tissues were directly homogenized in a 20 μL 3× SDS sample buffer using a motor-driven pestle. The same amounts of protein were subjected to SDS-polyacrylamide gel electrophoresis, and the separated proteins were transferred onto polyvinylidene fluoride membranes. The membranes were blocked for 1 h at 22°C with 5% milk or 5% FBS (for anti-pRab) in Tris-buffered saline containing 0.05% Tween20 (TBS-T) and then incubated overnight with primary antibodies at 4°C. After washing three times with TBS-T, the membranes were incubated with horseradish peroxidase (HRP)-conjugated secondary antibodies at 22°C for 1 h. After washing three times with TBS-T, signals were detected with Immobilon Forte Western HRP substrate (cat. no. WBLUF0500; Millipore, Merck, Darmstadt, Germany). The blot images were obtained using Fusion FX6 Edge (Vilber-Lourmat, Marne-la-Vallée, France).

#### Whole-mount immunostaining

Synapse boutons in larval motor neurons were visualized by whole-mount immunostaining as previously described.^35^ For image processing of synapse boutons in the A5 or A6 segment, 30 Z-stack images were taken at 0.35- to 0.70-μm intervals and reconstituted using ImageJ. The values of Arl8 aggregation-positive terminals in the graphs were calculated as the percentage of Arl8-accumulated terminals per total synaptic terminals in the same segments. Antibodies used in immunocytochemistry were anti-Arl8 [1:100; Developmental Studies Hybridoma Bank (DSHB), Arl8], anti-Rbsn5 (1:1000; gift from Dr. A. Nakamura), anti-Rab7 (1:100; DSHB, Rab7), anti-Calnexin 99A (1;100; DSHB, cnx99A 6-2-1), anti-Ref(2)P (1;100; in-house, 1532052), anti-LAMP1 (1:50; DSHB, H4A3); anti-INPP5F (1:200; in-house, GP-C2), anti-Aux (1:200; in-house, GP-1), anti-Chc (1:100; in house, AF19050743-001), anti-α-Synuclein (1:250; Abcam, MJFR1, ab138501), anti-α-Tubulin (1:200; Cell Signaling Technology, 11H10), anti-Tau (1:1000; Tau-C, gift from Dr. Y. Motoi), anti-GFP-FITC (1:500; Abcam, ab6662), Alexa Fluor594- (1:200), or DyLight649- (1:500) conjugated anti-HRP (123-585-021, Jackson ImmunoResearch, West Grove, PA). Secondary antibodies used were anti-rabbit Alexa Fluor Plus or Alexa Fluor (1:200; A32731 and A11012, Thermo Fisher Scientific, Waltham, MA), anti-mouse Alexa Fluor Plus or Alexa Fluor (1:200; A32723, A11017, and A11032, Thermo Fisher Scientific), and anti-guinea pig Alexa Fluor (1:200; A21435, Thermo Fisher Scientific).

#### TEM analysis and CLEM

Transmission electron microscopy (TEM) images were obtained using an electron microscope (HT7700, Hitachi High-Tech Corporation, Tokyo, Japan) at the Laboratory of Ultrastructural Research of Juntendo University as previously described.^79^ For correlative light-electron microscopy (CLEM), third instar larvae were dissected in HL-3 solution. Tissues were fixed with phosphate buffer containing 2.4% paraformaldehyde and 1% glutaraldehyde for 15 min. Synaptic boutons of motor neurons were labeled by whole-mount immunostaining as previously described^35^ and imaged on a Zeiss LSM880 Airyscan confocal microscopy (Oberkochen, Germany). After fluorescence imaging, TEM images were obtained using an electron microscope (HT7700).

#### Alr8 transport assay

Third-instar larvae were dissected in HL-3 medium containing 0.5 mM Ca^2+^, and axons of motor neurons were imaged each 0.6 s for 1 min on a Leica SP5 confocal microscope. Kymograph of Arl8-GFP was generated using the reslice tool in the Fiji ImageJ software and analyzed. Detailed protocols for sample preparation and data analysis can be found in.^80^

#### Electrophysiology

Third-instar larvae were dissected in HL-3, and mEJPs from NMJs were recorded using an electrophysiological setup equipped with an Eclipse FN1 microscope (Nikon, Tokyo, Japan), a Multiclamp 700B amplifier (Molecular Devices, San Jose, CA), and a Digidata 1550A data acquisition system (Molecular Devices). Dissected larvae were incubated in HL-3 containing 0.375 mM Ca^2+^, and a recording electrode filled with 3 M KCl was inserted into muscle 6 of the A3 or A4 segment containing NMJs. All data were analyzed using Mini-Analysis software (Synaptosoft, Fort Lee, NJ).

#### Climbing assay

For each genotype, two vials (25 flies/vial) were prepared. Vials (25 mm diameter × 180 mm height) were tapped gently on the table and left standing for 18 s. The number of flies that climbed at least 60 mm was recorded and represented as percentage.

#### α-Synuclein and Tau preparation and uptake assay

Recombinant α-Synuclein was purified from bacteria BL21(DE3) harboring pRK172-human α-synuclein using a Q Sepharose column (17051010, Cytiva, Tokyo, Japan).^79^ Recombinant Tau was purified from bacteria BL21(DE3) harboring pRK172-4R2N Tau using an SP Sepharose column (17072910, Cytiva).^81^ The third instar larvae were dissected in Schneider medium (cat. no. 21720024, Gibco, Thermo Fisher Scientific, Waltham, MA) with 10% fetal bovine serum. The motor neuron axon was sucked into a glass electrode and stimulated with 5 V, 1 Hz for 10 min in Schneider medium with 10 μM α-Synuclein or Tau. After stimulation, dissected larvae were incubated for 30 min in Schneider medium with 10 μM α-Synuclein or Tau and fixed with 4% paraformaldehyde in phosphate-buffered saline (PBS).

#### Histochemical analysis of human brain

Triple immunofluorescence staining was performed using 6-μm-thick, 4% paraformaldehyde-fixed, paraffin-embedded sections from the midbrain of clinically diagnosed and pathologically or genetically confirmed PD and control cases collected by the Department of Neurology, Juntendo University Hospital. Deparaffinized sections microwaved in Tris EDTA buffer, pH 9.0 (S2367, Agilent-DAKO, Santa Clara, CA) for 10 min for antigen retrieval, were neutralized with PBS. Sections were treated with blocking buffer (cat. no. 0634964, Blocking One Histo, Nacalai Tesque) and incubated with anti-Arl8A (1:50; HPA038759, Atlas) or anti-Arl8B (1:50; 13049-1-AP, Proteintech, Rosemont, IL) antibodies along with anti-α-Synuclein (1:250; pSyn#64, FUJIFILM Wako) and anti-Tyrosine hydroxylase (1:1000; ab76442, Abcam) overnight at 4°C. Primary antibodies were visualized with secondary antibodies (1: 1,000, Alexa Fluor405, 488, 594, and 647, Thermo Fisher Scientific) for 1 h at RT. To reduce lipofuscin autofluorescence, sections were further treated with TrueBlack Lipofuscin Autofluorescence Quencher (cat. no. 23007, Biotium, Inc. Fremont, CA), diluted with 70% ethanol for 30 s, and briefly washed with PBS before mounting with VECTASHIELD mounting medium (H1800, Vector laboratories, Newark, CA) and assessed using LSM880 with the Airyscan laser-scanning microscope system (Zeiss).

### Quantification and statistical analysis

All data in bar graphs were expressed as mean ± SEM and graphs were generated using JMP v11.0.0 (SAS Institute Cary, NC). p values less than 0.05 were considered statistically significant. Student t-test or Dunnett’s test was used to determine significant differences between two specific groups or between multiple groups of interest. The data distribution was assumed to be normal, although this assumption was not formally tested. Randomization was used in each genotype. Counting of the vesicles in the electron micrographs in Figure 6C was performed blindly by TI, J-YL, and C. Cui. Other data and analyses were not performed blind to the conditions of the experiments. All of the statistical details of the experiments can be found in the figure legends.

## Data Availability

• All data reported in this paper will be shared by the lead contact upon request. • This paper does not report original code. • Any additional information required to reanalyze the data reported in this paper is available from the lead contact upon request.

## Supplemental information

Document S1. Figures S1–S7 and Tables S1

Table S2. Genotypes used in Figures, related to STAR Methods
