# Supplementary material for: Parkinson disease-associated Leucine-rich repeat kinase regulates UNC-104-dependent axonal transport of Arl8-positive vesicles in Drosophila
Source: iScience. 2022 Nov 2;25(12):105476. doi: 10.1016/j.isci.2022.105476 (PMC9672966; doi:10.1016/j.isci.2022.105476)
Supplement: Document S1. Figures S1–S7 and Tables S1 [file mmc1.pdf]

iScience, Volume *25*

Supplemental information

Parkinson disease-associated Leucine-rich repeat kinase regulates UNC-104-dependent axonal transport of Arl8-positive vesicles in *Drosophila*

Tsuyoshi Inoshita, Jun-Yi Liu, Daisuke Taniguchi, Ryota Ishii, Kahori Shiba-Fukushima, Nobutaka Hattori, and Yuzuru Imai

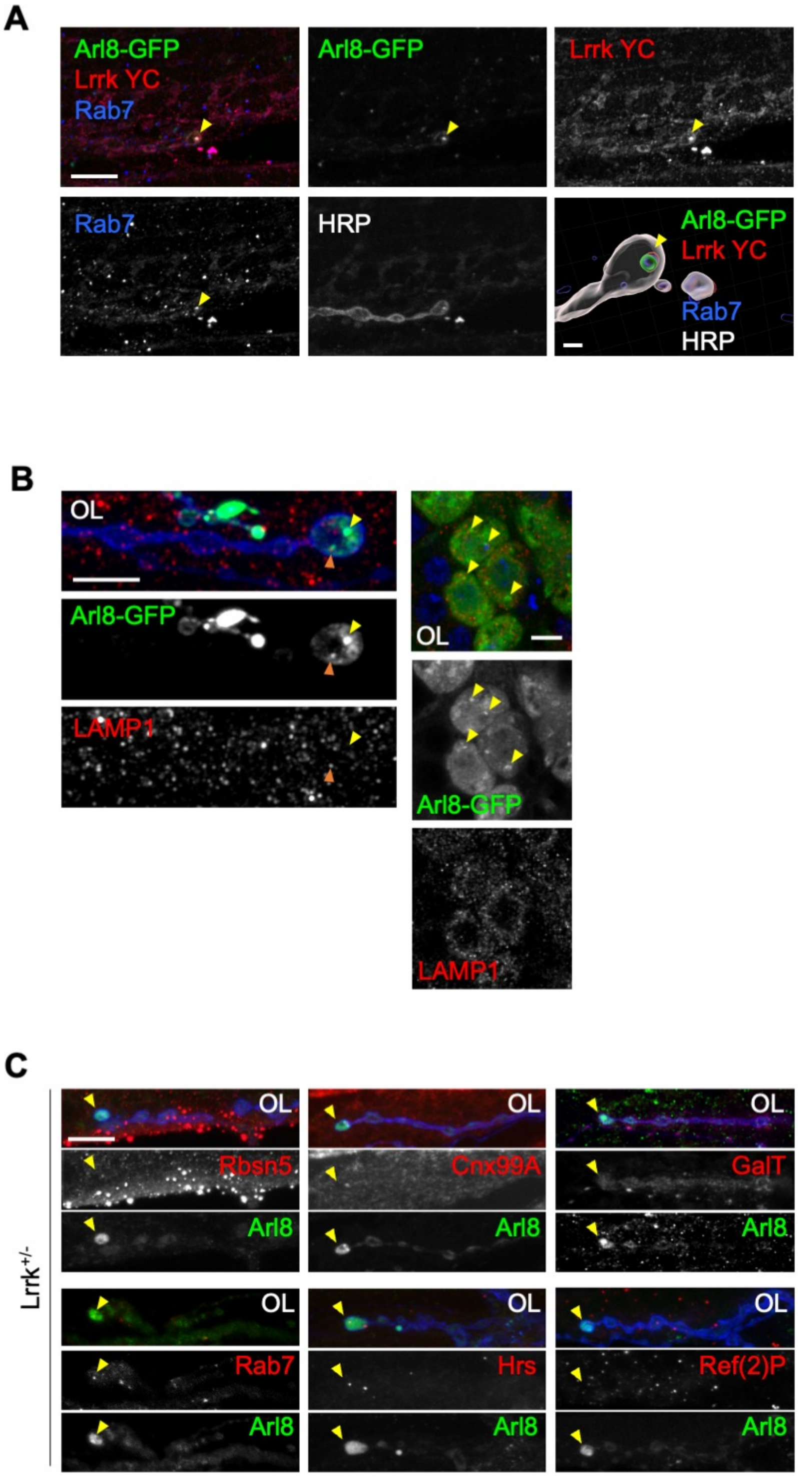

Figure S1. Arl8 aggregates do not contain the endoplasmic reticulum, *trans*-Golgi, and autophagy-associated Ref(2)P, Related to Figure 1.

(**A**) Co-localization of Arl8, Lrrk Y1383C and Rab7 in the synaptic boutons. Three-dimensional image was constructed using the surface tool of Imaris software (right bottom). Scale bars, 20 μm (top) and 2 μm (right bottom).

(**B**) Localization of Arl8 and LAMP1 in the synaptic boutons (left) and cell bodies (right). Lrrk Y1383C transgene was expressed using the *D42-GAL4 driver*. Orange and yellow arrowheads indicate Arl8 aggregates with LAMP1 and without LAMP1, respectively. Each top image shows overlay (OL), while the middle and bouton images show single-channel images in grayscale. Scale bars, 5 μm.

(**C**) Localization of Rabenosyn-5 (Rbsn5, early endosome marker), Calnexin (Cnx99A, endoplasmic reticulum marker), galactosyltransferase (GalT, *trans*-Golgi marker), Rab7 (late endosome marker), Hrs (multivesicular body maker), and Ref(2)P (autophagy marker) in Arl8-positive boutons of *LRRK*$^{+/-}$. Synaptic boutons were visualized with DyLight649-conjugated anti-HRP (blue). Arrowheads indicate Arl8 aggregates. *trans*-Golgi was visualized with *UAS-hGalT-TagRFP*, and others were visualized using antibodies. Scale bar, 10 μm.

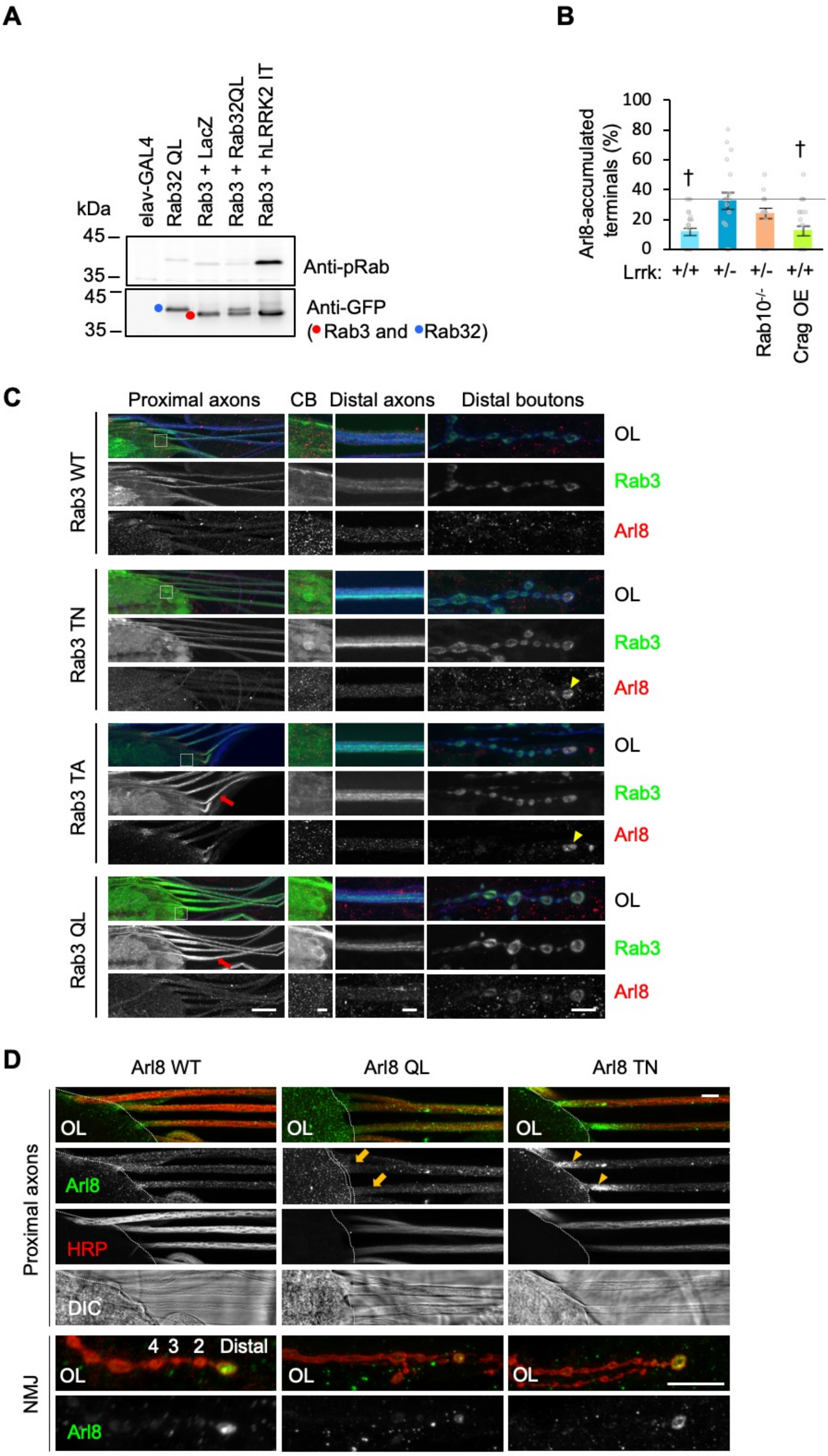

A
kDa
elav-GAL4
Rab32 QL
Rab3 + LacZ
Rab3 + Rab32QL
Rab3 + hLRRK2 IT
45
35
45
35
Anti-pRab
Anti-GFP
(Rab3 and Rab32)
B
Arl8-accumulated terminals (%)
100
80
60
40
20
0
†
†
Lrrk:
+/+
+/-
+/-
+/+
Rab10-/-
Crag OE
C
Proximal axons
CB
Distal axons
Distal boutons
Rab3 WT
Rab3 TN
Rab3 TA
Rab3 QL
OL
Rab3
Arl8
D
Arl8 WT
Arl8 QL
Arl8 TN
Proximal axons
OL
Arl8
HRP
DIC
NMJ
4
3
2
Distal

Figure S2. Distribution of the GDP/GTP forms of Arl8 in the NMJs or axons, Related to Figure 2.

(**A**) Rab32 Q79L does not stimulate Rab3 phosphorylation. YFP-tagged Rab3 and/or YFP-Rab32Q79L transgenes were expressed using the *elav-GAL4 driver*. LacZ and hLRRK2 I2020T served as negative and positive controls, respectively.

(**B**) Rab10 activity does not affect Arl8 accumulation. Crag is Rab10 GEF. Graph represents mean ± SEM (n = 18-26 NMJs in 6-7 flies). † $p < 0.05$ *vs.* $\mathrm{Lrrk}^{+/-}$ by Dunnett's test.

(**C**) Rab3 GTPase activity influences its neuronal distribution. Distribution of Rab3 in the cell body and the proximal and distal axons of $Lrrk^{+/+}$ larval motor neurons. Red arrows indicate Rab3 enrichment at the proximal axons. Yellow arrowheads indicate Arl8 aggregates at the distal boutons. CB, high-magnification images of cell bodies in the boxes of the left images. Scale bars, 20 µm.

(**D**) Distribution of Arl8 WT, Q75L (QL), and T34N (TN) in the proximal axons and the NMJs on w^{1118} genetic background. Arl8 TN is enriched in the proximal axons (arrowheads), while Arl8 QL is not enriched in this region (arrow). Accumulation of Arl8 in the terminal bouton (Distal) is more prominent in Arl8 WT than in Arl8 QL and Arl8 TN. DIC, differential interference contrast images. Scale bars, 20 µm.

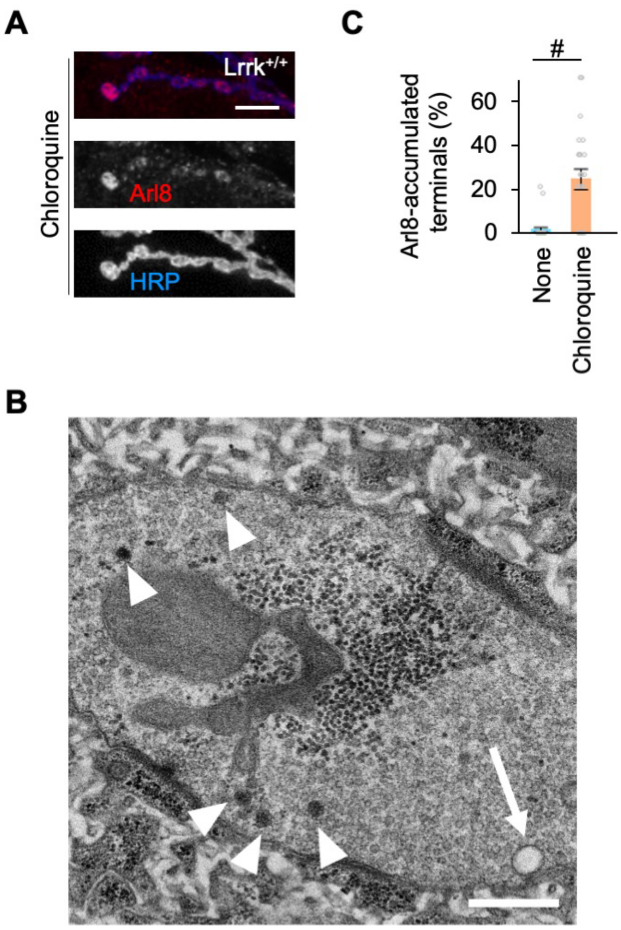

Figure S3. Chloroquine induces Arl8 accumulation in the boutons, Related to Figure 3.

(**A**) Distribution of Arl8 in *Lrrk*$^{+/+}$ NMJs reared on diets containing 2.5 mg/ml chloroquine. Scale bar, 20 μm.

(**B**) Ultrastructure of *Lrrk*$^{+/+}$ synaptic boutons treated as in (**A**). Arrowheads and an arrow indicate DCVs and a large vesicle, respectively. Scale bar, 500 nm.

(**C**) Graph (mean ± SEM) represents Arl8-accumulated boutons in *Lrrk*$^{+/+}$ NMJs (n = 23-26 NMJs in 6-7 flies). # $p < 0.05$ by two-tailed t-test.

A

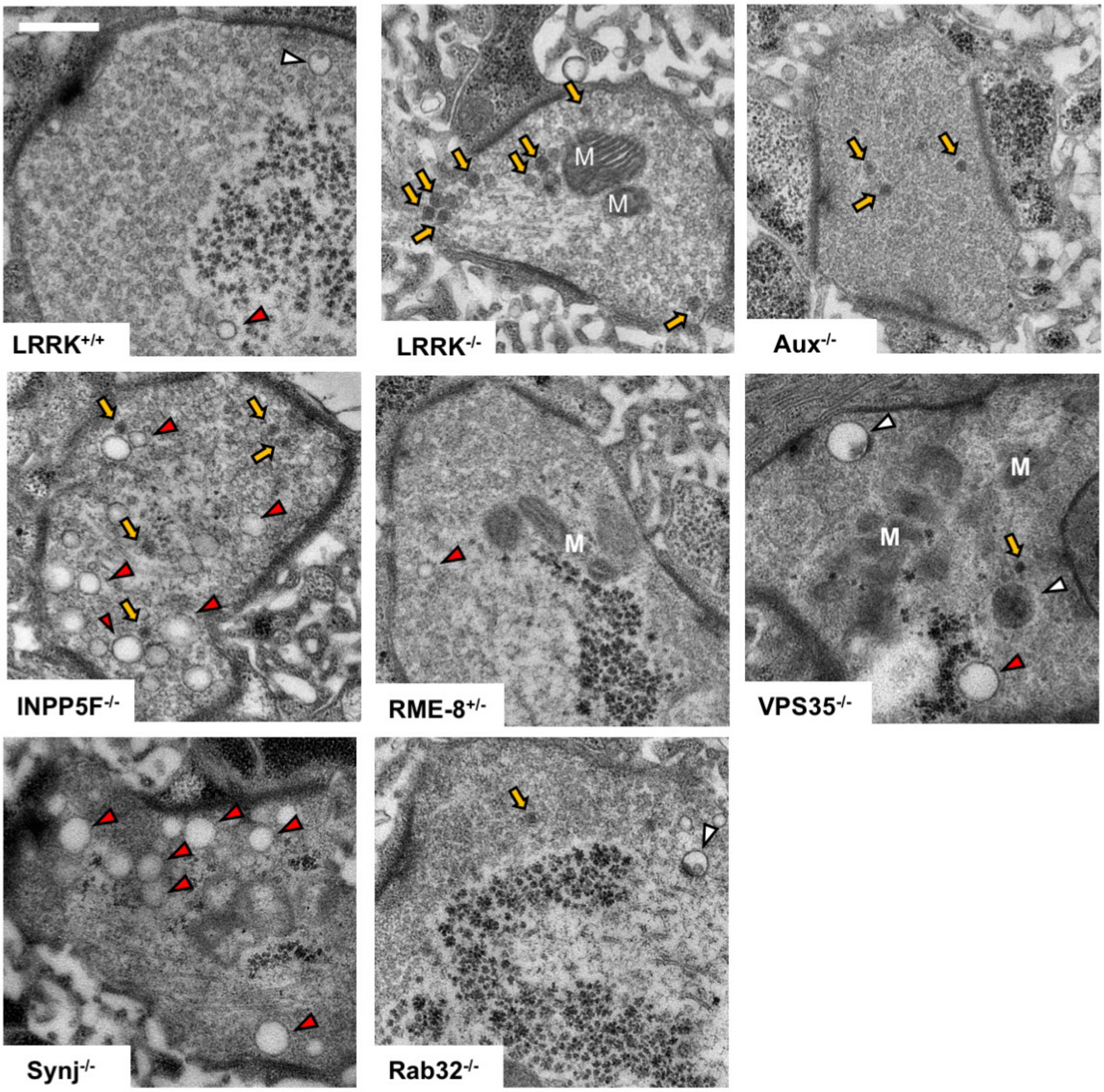

B

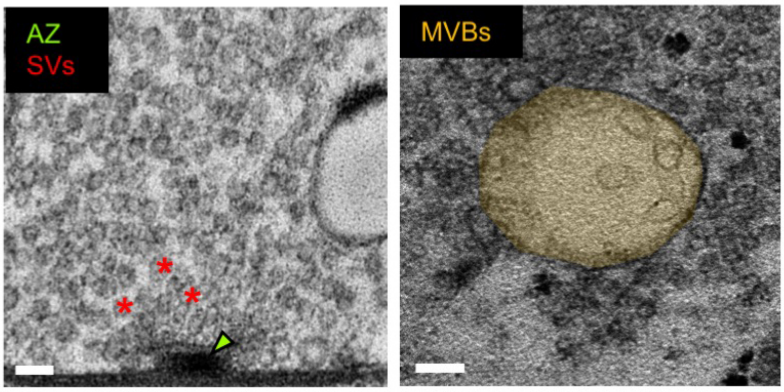

Figure S4. Presynaptic phenotypes by loss-of-function mutations of PD genes, Related to Figure 6.

(**A**) Ultrastructure of synaptic boutons with the indicated genotypes. Orange arrows and red arrowheads indicate DCVs and large vesicles, respectively. White arrowheads indicate multivesicular bodies (MVBs). M, mitochondrion. Scale bar, 500 nm.

(**B**) Definition of vesicle numbers. The number of DCVs, SVs (red asterisks), large vesicles, and MVBs in a 500-nm square area containing an active zone (AZ, green arrowhead) was counted as the vesicle numbers in Figure 6. MVBs (orange) are unilamellar vesicles with inner vesicles. Scale bars, 100 nm.

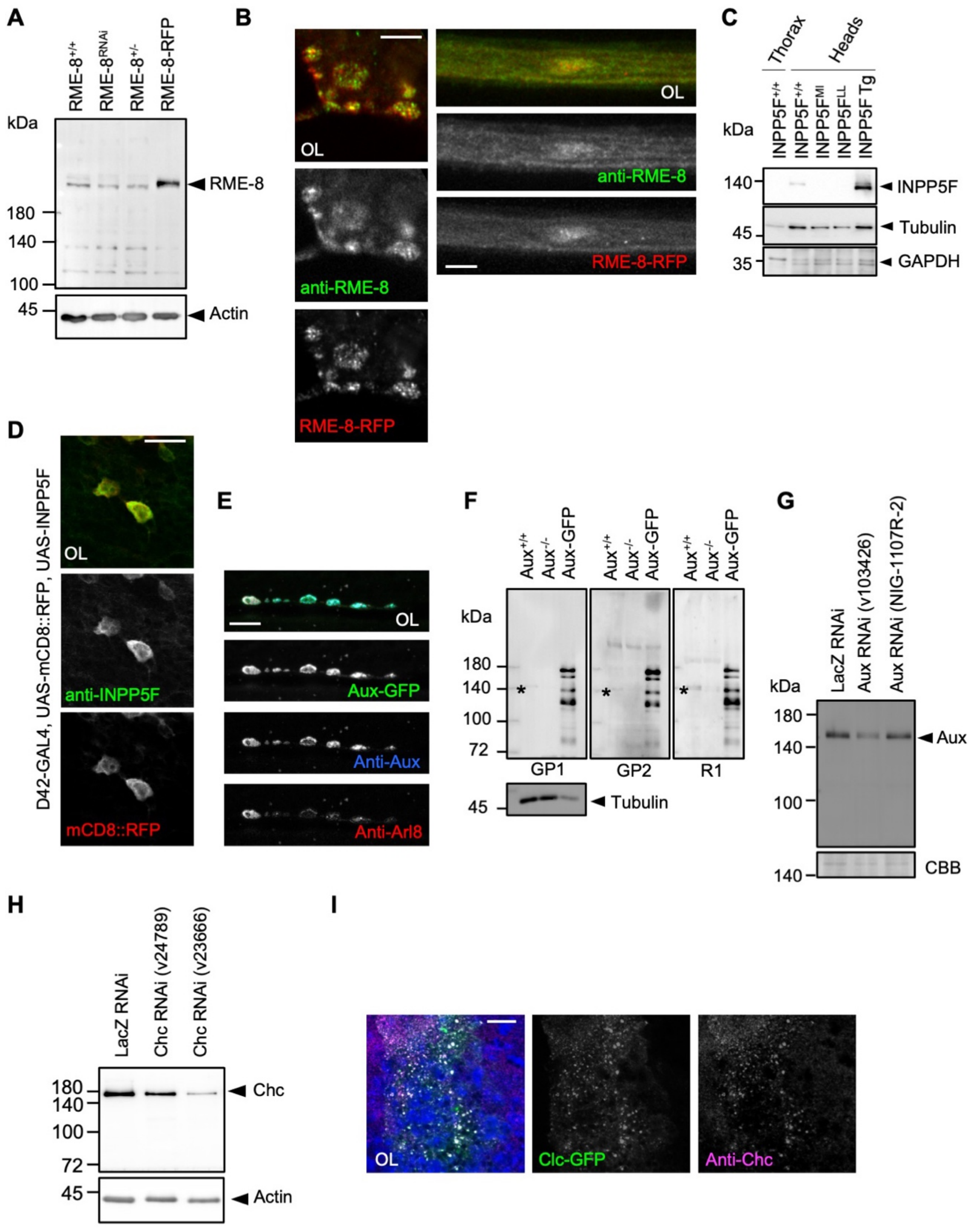
A
RME-8+/+
RME-8RNAi
RME-8+/-
RME-8-RFP
kDa
RME-8
180
140
100
45
Actin
B
OL
anti-RME-8
RME-8-RFP
C
Thorax
Heads
INPP5F+/+
INPP5FMI
INPP5FLL
INPP5F Tg
INPP5F
Tubulin
GAPDH
140
45
35
D
D42-GAL4, UAS-mCD8::RFP, UAS-INPP5F
anti-INPP5F
mCD8::RFP
E
Aux-GFP
Anti-Aux
Anti-Arl8
F
Aux+/+
Aux-/-
Aux-GFP
GP1
GP2
R1
Tubulin
72
G
LacZ RNAi
Aux RNAi (v103426)
Aux RNAi (NIG-1107R-2)
Aux
CBB
H
Chc RNAi (v24789)
Chc RNAi (v23666)
Chc
I
Clc-GFP
Anti-Chc

Figure S5. Validation of antibodies generated in this study, Related to Figure 7.

(**A**) Validation of anti-RME-8 antibody using adult heads of the *RME-8*$^{+/-}$ (*RME-8*^{D3}), *RME-8 RNAi*, and *RME-8-RFP* lines. *RME-8 RNAi* (v107706) and *RME-8-RFP* were expressed using the *elav-GAL4* driver.

(**B**) Validation of anti-RME-8 antibody for histochemical analysis. Co-localization of anti-RME-8 immunosignals and RME-8-RFP in the NMJs (left) and axons (right) of larval motor neurons. Scale bars, 5 μm (left) and 10 μm (right).

(**C**) Validation of guinea pig anti-INPP5F antibody (GP2-C2) using adult heads with the indicated genotypes. *w*^{1118} was used as *INPP5F*$^{+/+}$. INPP5F transgene (Tg) was expressed using the *Da-GAL4* driver.

(**D**) Validation of anti-INPP5F antibody (GP2-C2) in the larval ventral ganglion co-expressing mCD8::RFP and INPP5F. Scale bar, 10 μm.

(**E**) Validation of anti-Aux antibody for histochemical analysis. Co-localization of anti-Aux immunosignals (blue) with GFP signals (green) and anti-Arl8 immunosignals (red) in the *Lrrk*$^{+/-}$ NMJs expressing Aux-GFP using the *elav-GAL4* driver. Scale bar, 5 μm.

(**F**) Validation of guinea pig (GP1 and GP2) and rabbit (R1) anti-Aux antibodies using adult heads of *Aux*$^{-/-}$ (*Auxilin*$^{727/I670K}$) and Aux-GFP lines. Aux-GFP was expressed using the *elav-GAL4* driver. Asterisks indicate endogenous Aux.

(**G**) Validation of anti-Aux antibodies using adult heads of *Aux RNAi* lines. *Aux RNAi* (strong, v103426 and weak, NIG-1107R-2) were expressed using the *elav-GAL4* driver and Aux expression was analyzed in the adult brain with anti-Aux (R1).

(**H**) Validation of anti-Chc antibody using adult heads of *Chc RNAi* lines. *Chc RNAi* (v24789 and v23666) were expressed using the *elav-GAL4* driver.

(**I**) Validation of anti-Chc antibody for histochemical analysis. Co-localization of anti-Chc immunosignals with clathrin light chain (Clc)-GFP in the larval wing discs counter-stained with DAPI (blue). Clc-GFP was expressed using *Dpp-GAL4*. Scale bar, 5 μm.

A

	Accumulation with Arl8	LOF (a	LOF (b in Lrrk$^{+/-}$	OE in Lrrk$^{+/-}$	OE in Lrrk$^{-/-}$
RME-8	**+**	**–**		**Suppress**	
INPP5F	**+**	**+**	**Mildly augment**	**Suppress**	
Auxilin	**+/-**	**–**	**Augment**	**Augment**	**Augment**
VPS35	**–**	**+**		**Suppress**	
Synj	**–**	**–**			
Rab32	**–**	**–**		**Augment**	**N.D.**
EndoA	**+/-**	**N.D.**	**N.D.**	**N.D.**	**N.D.**

a) Heterozygous loss-of-function alleles were used in all genotypes except for Auxilin.
b) Heterozygous loss-of-function alleles were used in all genotypes.

B

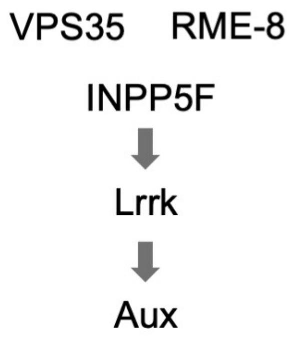

Figure S6. PD-related genes that modulate Arl8 accumulation, Related to Figure 7.

(**A**) Gene list analyzed in this study. Accumulation with Arl8; co-localization analysis using specific antibodies or fluorescent protein-tagged proteins; LOF, Arl8 phenotype in LOF mutants for the indicated genes. RME-8 was analyzed in a heterozygous mutant due to the early lethality of the homozygous mutant. OE, neuronal overexpression of the indicated genes by *elav-GAL4*. N.D., not determined.

(**B**) Genetic positional relationship of PD-related genes with *Lrrk* inferred from the Arl8 phenotype.

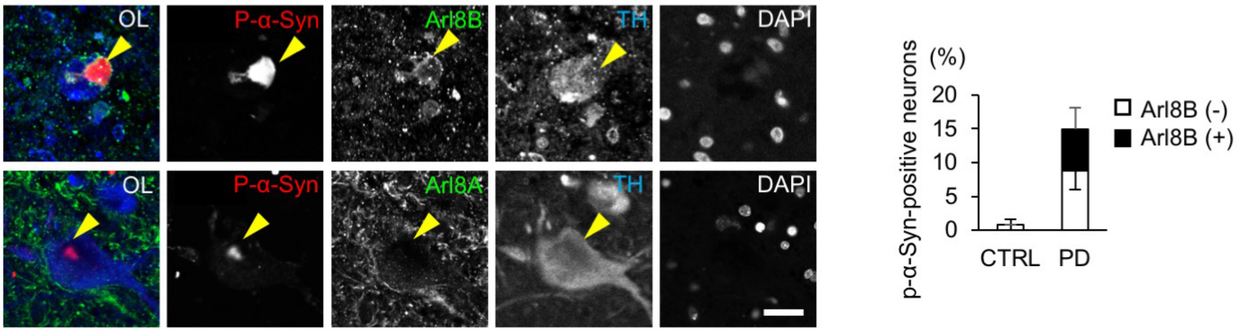

Figure S7. Arl8B, but not Arl8A, is partially co-localized with Lewy bodies, Related to Figure 8.

Lewy bodies (arrowheads) in the midbrain dopaminergic neurons of PD patients. (Upper) Arl8B-immunosignals localized around phospho-Ser129 α-Synuclein-positive Lewy bodies (P-α-Syn) in the midbrain dopaminergic neurons. (Lower) The absence of Arl8A-immunosignals in Lewy bodies. Tyrosine hydroxylase (TH) was used as a dopaminergic neuron marker. DAPI staining visualized nuclei. Scale bar, 20 μm. Graph represents the percentage (mean ± SEM) of TH-positive neurons containing phospho-α-Synuclein-positive Lewy bodies (with anti-Arl8B-positive and negative) (n = 6 cases in each). CTRL, Control.

Table S1. Clinical information of human samples used in this study, Related to STAR Methods.

	Age (years)	Sex	Disease duration (years)	Pathological diagnosis
Control	85	Female	N/A	Thalamic hemorrhage
Control	68	Female	N/A	Cerebral infarction
Control	68	Male	N/A	Cerebral infarction
Control	72	Male	N/A	Primary age-related tauopathy
Control	79	Male	N/A	Chronic ischemic changes
Control	50	Male	N/A	Cerebral infarction
PD	88	Male	2	Lewy body disease
PD	86	Male	5	DLBD common form (Lewy body disease + AD pathology)
PD	65	Male	17	Lewy body disease
PD	69	Male	7	DLBD common form (Lewy body disease + AD pathology)
PD	83	Female	6	Lewy body disease
PD	74	Male	11	DLBD common form (Lewy body disease + AD pathology)

AD, Alzheimer's disease; DLBD, diffuse Lewy body disease
